# Primary cilia mediate early life programming of adiposity through lysosomal regulation in the developing mouse hypothalamus

**DOI:** 10.1038/s41467-020-19638-4

**Published:** 2020-11-13

**Authors:** Chan Hee Lee, Do Kyeong Song, Chae Beom Park, Jeewon Choi, Gil Myoung Kang, Sung Hoon Shin, Ijoo Kwon, Soyoung Park, Seongjun Kim, Ji Ye Kim, Hong Dugu, Jae Woo Park, Jong Han Choi, Se Hee Min, Jong-Woo Sohn, Min-Seon Kim

**Affiliations:** 1 Asan Institute for Life Science, University of Ulsan College of Medicine, Seoul, 05505 Korea; 2 Division of Endocrinology and Metabolism, Department of Internal Medicine, Asan Medical Center and University of Ulsan College of Medicine, Seoul, 05505 Korea; 3 Department of Biomedical Science, Asan Medical Institute of Convergence Science and Technology, Asan Medical Center and University of Ulsan College of Medicine, Seoul, 05505 Korea; 4 Department of Biological Sciences, Korea Advanced Institute of Science and Technology, Daejeon, 34141 Korea; 5 Developmental Neuroscience & Diabetes and Obesity Programs, Children’s Hospital Los Angeles, University of Southern California, Los Angeles, CA 90027 USA

**Keywords:** Cellular neuroscience, Neuronal development, Obesity

## Abstract

Hypothalamic neurons including proopiomelanocortin (POMC)-producing neurons regulate body weights. The non-motile primary cilium is a critical sensory organelle on the cell surface. An association between ciliary defects and obesity has been suggested, but the underlying mechanisms are not fully understood. Here we show that inhibition of ciliogenesis in POMC-expressing developing hypothalamic neurons, by depleting ciliogenic genes IFT88 and KIF3A, leads to adulthood obesity in mice. In contrast, adult-onset ciliary dysgenesis in POMC neurons causes no significant change in adiposity. In developing POMC neurons, abnormal cilia formation disrupts axonal projections through impaired lysosomal protein degradation. Notably, maternal nutrition and postnatal leptin surge have a profound impact on ciliogenesis in the hypothalamus of neonatal mice; through these effects they critically modulate the organization of hypothalamic feeding circuits. Our findings reveal a mechanism of early life programming of adult adiposity, which is mediated by primary cilia in developing hypothalamic neurons.

## Abstract (web summary)

Ciliary defects and obesity has been associated, but the underlying mechanism is unclear. Here, the authors show that inhibition of ciliogenesis in POMC neurons during development results in lysosomal protein degradation-dependent axonal disruption and adult obesity in mice.

## Introduction

Obesity has become a leading health problem across the globe^1^. Human epidemiology studies have demonstrated a strong association between maternal nutrition/birth weights and the adulthood risks of obesity-related medical comorbidity^2,3^. In rodent experiments, both overnutrition and undernutrition during gestation and lactation predispose the offspring to obesity and metabolic disorders in later life^4,5^. These epidemiological and experimental observations suggest that nutritional conditions in early life have lifelong impacts on body energy metabolism by programing it to adapt to the altered nutritional environments.

The hypothalamus is a critical organ for maintaining energy balance and determining adiposity^6^. Proopiomelanocortin (POMC) is the precursor peptide for the α-melanocyte-stimulating hormone (αMSH) that induces a negative energy balance upon binding to the central melanocortin 4 receptors (MC4Rs)^7^. POMC-producing neurons in the hypothalamic arcuate nucleus (ARH) are regarded as a representative catabolic neuron as their loss leads to obesity^8^. In rodents, POMC-expressing neuronal progenitors emerge in the hypothalamic ventricular zone at embryonic day (E) 10.5–11.5^9^. They subsequently expand their pool and differentiate to either POMC or non-POMC neurons^10^. ARH POMC neurons are unwired at birth, but they establish neural circuits during the lactation period by projecting axons to intra- and extra-hypothalamic areas^11^. Of note, the circulating levels of adipocyte-derived hormone leptin rise during the early postnatal period and triggers axonal projections of ARH neurons^12^. By contrast, maternal overnutrition and undernutrition repress these processes via yet unknown mechanisms.

Most mammalian cells including hypothalamic neurons, have tiny hair-like primary cilia on their cell surfaces^13,14^. To generate these cilia, ciliary structural proteins are transported to the ciliary tip via a process known as antegrade intraflagellar transport (IFT), which is mediated by kinesin-II motor and IFT complex B^15^. Disruption of this process inhibits cilia formation^16,17^. Primary cilia were once thought to be an evolutionary rudiment but are now regarded as a pivotal signaling organelle^18^. During development, the primary cilia transduce sonic hedgehog (SHH) signaling, which is a critical regulator of the ventral patterning of neuronal tube^19^. A lack of cilia in cerebellar and hippocampal neuronal precursors limits neurogenesis, leading to hypotrophy of the cerebellum and hippocampus^20,21^. Primary cilia in adult-born hippocampal neurons and GABAergic interneurons are involved in dendritic refinement and synaptic formation^22,23^. However, the role of the primary cilia during hypothalamic development remains elusive.

Several lines of evidence have documented a connection between primary cilia and obesity^24^. Human genetic ciliopathy such as Bardet–Biedl syndrome and Alström’s syndrome manifests obesity and type 2 diabetes^25,26^. Generalized ciliary dysgenesis in adult mice leads to hyperphagic obesity^27^. Disruption of cilia formation specific to the synapsin 1- or POMC-expressing neurons also causes obesity albeit to a lesser degree, indicating the contribution of neuronal cilia in obesity development^27^. Moreover, neuropeptide receptors involved in energy metabolism such as the MC4R, melanin concentrating hormone receptor 1, and neuropeptide Y receptor 2, are expressed on the primary cilia of a subset of hypothalamic neurons^28–30^. Hence, the primary cilia in hypothalamic neurons may serve as a signaling platform for neuropeptides or hormones whereby they contribute to the homeostatic control of energy metabolism^31^.

In our present study, we have uncovered an important role of the primary cilia in the neuronal circuit development of ARH POMC neurons. We also provide evidence that the early life nutritional and hormonal environments impact adulthood adiposity by regulating ciliogenesis in the developing hypothalamic neurons.

## Results

### Inhibition of ciliogenesis in the developing POMC neurons, but not in adult POMC neurons, causes adulthood obesity

**Fig. 1:**
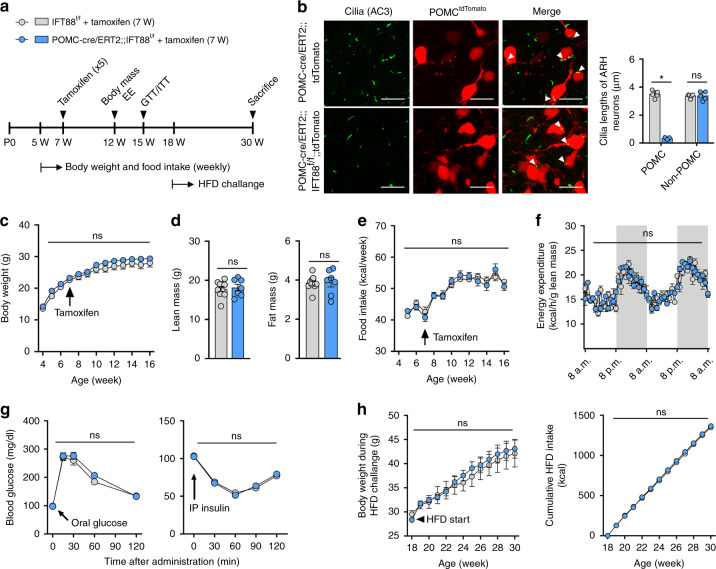
Adult-onset ciliary dysgenesis in POMC neurons does not change energy and glucose metabolism. **a** Experimental scheme in mice with POMC-specific IFT88 depletion that was induced by tamoxifen injections at 7 weeks. **b** Representative confocal images of AC3 (adenylyl cyclase-3, cilia marker) and tdTomato double staining in the ARH of POMC-cre/ERT2;;IFT88^f/f^;;tdTomato mice and POMC-cre/ERT2;;tdTomato mice (*n* = 5). Arrowheads indicate cilia of POMC^tdTomato^ neurons. The graph depicts the average cilia lengths of about 100 POMC and non-POMC neurons. *p < 0.001. Scale bars: 20 μm. **c** Body weights in POMC-cre/ERT2;;IFT88^f/f^ mice and their IFT88^f/f^ littermates on a chow diet (*n* = 10 for IFT88^f/f^ mice, *n* = 8 for POMC-cre/ERT2;;IFT88^f/f^ mice). **d** Lean mass and fat mass measured at 12 weeks (*n* = 10 for IFT88^f/f^ mice, *n* = 7 for POMC-cre/ERT2;;IFT88^f/f^ mice). **e** Average weekly food intake during 4–16 weeks (*n* = 5). **f** Energy expenditure measured at 12 weeks (*n* = 5). **g** Glucose and insulin tolerance tests performed at 15 weeks (*n* = 7). **h** Body weights and cumulative food intake during a high-fat diet (HFD) challenge (*n* = 7). Data are presented as the mean ± SEM values. Statistics were performed using two-sided Student’s *t* test (**b**, **d**) and one-sided two-way ANOVA (**c**, **e**–**h**) followed by post hoc least significant difference (LSD) test. ns not significant.

The induction of defective ciliogenesis in POMC neurons, through the depletion of a critical ciliogenic component the kinesin-II motor subunit KIF3A^17^ using cre-lox recombination, induces obesity and hyperphagia^27^. Given the wide expression of POMC in immature ARH neurons during hypothalamic development^10^, the obesity phenotype in POMC-cre;;KIF3A^f/f^ mice may be the result of ciliary defects in either POMC-expressing neural progenitors or adult POMC neurons. To clarify this issue, we generated the mice with aberrant ciliogenesis specifically in adult POMC neurons. For this, we crossed POMC-cre/ERT2 mice with another important ciliogenic gene IFT88-floxed mice and injected tamoxifen to induce gene knockout at 7 weeks (Fig. 1a). Aberrant ciliogenesis was confirmed by staining cilia with an antibody against adenylyl cyclase-3 (AC3) as it is enriched in primary cilia of the central nervous system^32^. Stunted cilia were observed in the tdTomato-labeled POMC neurons of POMC-cre/ERT2;;IFT88^f/f^;;tdTomato mice but ciliogenesis in non-POMC neurons was unaltered (Fig. 1b). Successful cre-lox recombination was also confirmed by examining tdTomato expression in >90% of β-endorphin (β-END)-expressing POMC neurons (Supplementary Fig. 1a).

Notably, adult-onset ciliary dysgenesis in the POMC neurons did not induce significant changes in body weight, fat mass, or lean mass in male mice until 16 weeks (Fig. 1c, d). In addition, food intake, energy expenditure (EE), and glucose/insulin tolerance were not significantly altered in these cilia mutants (Fig. 1e–g). Moreover, POMC-cre/ERT2;;IFT88^f/f^ mice showed comparable weight gains and calorie intake to wild littermates upon a high-fat diet (HFD) feeding (Fig. 1h). These data suggested that adult-onset ciliary dysgenesis in POMC neurons does not significantly disrupt energy balance and that developmental defect may underlie POMC cilia-related obesity.

**Fig. 2:**
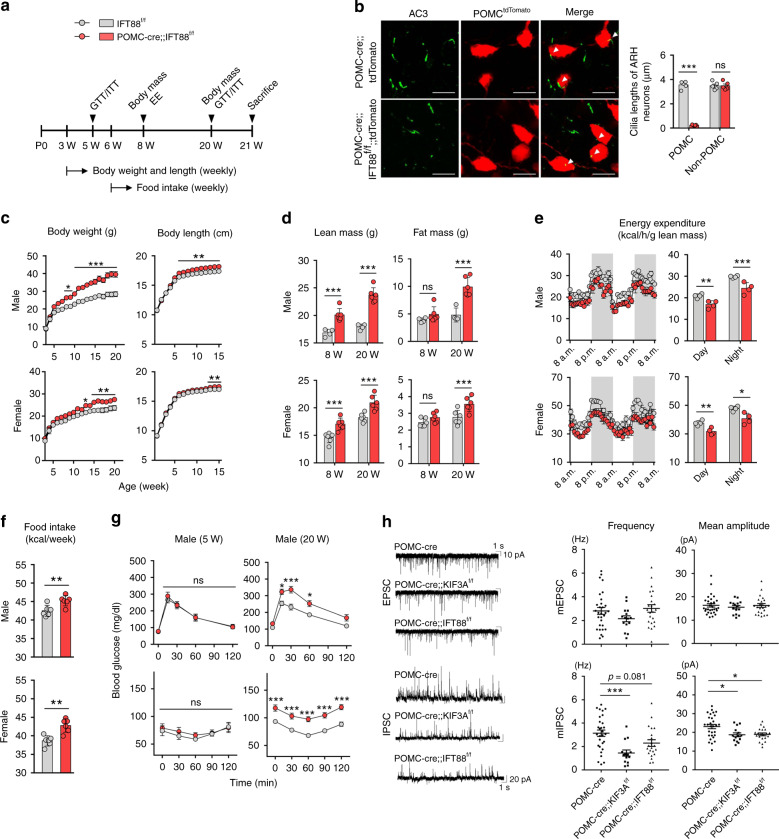
Inhibition of ciliogenesis in the developing POMC neurons causes obesity and glucose dysregulation in adulthood. **a** Experimental scheme in mice with POMC-specific IFT88 depletion that commenced from E11–E12. **b** Representative cilia images and measurement of cilia lengths in the ARH of POMC-cre;;IFT88^f/f^;;tdTomato mice and age-matched POMC-cre;;tdTomato mice (*n* = 5). Arrowheads indicate cilia of POMC^tdTomato^ neurons. Scale bars: 20 μm. **c** Body weights and body lengths of POMC-cre;;IFT88^f/f^ and IFT88^f/f^ male and female mice (body weights: *n* = 6 for POMC-cre;;IFT88^f/f^ males and *n* = 4 for the other 3 groups, body lengths: *n* = 7). **d** Lean mass and fat mass measured at 8 and 20 weeks (*n* = 4 for IFT88^f/f^ males, *n* = 6 for the other 3 groups). **e** Energy expenditure determined at 8 weeks (*n* = 4). **f** Average values of weekly food intake during 6–20 weeks (*n* = 6). **g** Glucose and insulin tolerance tests performed at 5 and 20 weeks (*n* = 5). **h** Patch clamp recordings showing the frequency and mean amplitude of the miniature excitatory postsynaptic currents (mEPSCs) and the miniature inhibitory postsynaptic currents (mIPSCs) in the POMC neurons lacking KIF3A or IFT88 (EPSC recording: *n* = 30 for POMC-cre mice, *n* = 15 for POMC-cre;;KIF3A^f/f^ mice, *n* = 23 for POMC-cre;;IFT88^f/f^ mice; IPSC recording: *n* = 29 for POMC-cre mice, *n* = 14 for POMC-cre;;KIF3A^f/f^ mice, *n* = 21 for POMC-cre;;IFT88^f/f^ mice). Data are presented as the mean ± SEM values. Statistics were performed using two-sided Student’s *t* test (**b**, **d**–**f**), one-sided two-way ANOVA (**c**, **g**) and one-sided one-way ANOVA (**h**) followed by post hoc LSD test and. *p < 0.05, **p < 0.01, ***p < 0.001 vs. IFT88^f/f^ controls. ns not significant.

To next evaluate the possible developmental role of the primary cilia in POMC-expressing neurons or neural precursors, we generated POMC-cre;;IFT88^f/f^ mice by mating POMC-cre mice and IFT88-floxed mice and performed metabolic phenotyping in these mice (Fig. 2a). In this model, the disruption of ciliogenesis would commence at around E10–E11^9^. First, successful disruption of ciliogenesis was confirmed by short stunted cilia in the POMC-tdTomato cells of POMC-cre;;IFT88^f/f^;;tdTomato mice (Fig. 2b). POMC-cre;;IFT88^f/f^ mice showed greater weight gain and accelerated linear growth compared to the IFT88^f/f^ littermates (Fig. 2c). Body composition analysis revealed that POMC-cre;;IFT88^f/f^ male and female mice had higher lean mass but unaltered fat mass at 8 weeks but increased lean and fat mass at 20 weeks (Fig. 2d). This data indicated that ciliary defect in POMC-expressing cells from the mid-embryonic period accelerates body growth and causes maturity-onset obesity. In addition, POMC-cre;;IFT88^f/f^ mice expended less energy and consumed more food compared to wild controls (Fig. 2e, f). They were glucose intolerant and insulin resistant at 20 weeks but not at 5 weeks (Fig. 2g). Therefore, increased energy intake and reduced expenditure contribute to the progression of obesity and altered glucose metabolism may be secondary to obesity. These metabolic phenotypes of POMC-cre;;IFT88^f/f^ mice largely recapitulated those of MC4R-deficient mice^33^, implying a reduced hypothalamic melanocortinergic tone.

Patch clamp recordings of POMC neurons revealed no alteration in the frequency or mean amplitude of the miniature excitatory postsynaptic currents (mEPSCs; Fig. 2h). By contrast, the mean amplitude and frequency of the miniature inhibitory postsynaptic current (mIPSC) was reduced or tended to be lower in ARH POMC neurons lacking KIF3A or IFT88 (Fig. 2h). The observed changes in the inhibitory synaptic activity suggested alterations in the synaptic input organization of POMC neurons but could not provide a simple explanation for the obesity phenotypes in POMC cilia mutants.

### Early postnatal ciliogenesis in POMC neurons influences energy metabolism in adulthood

**Fig. 3:**
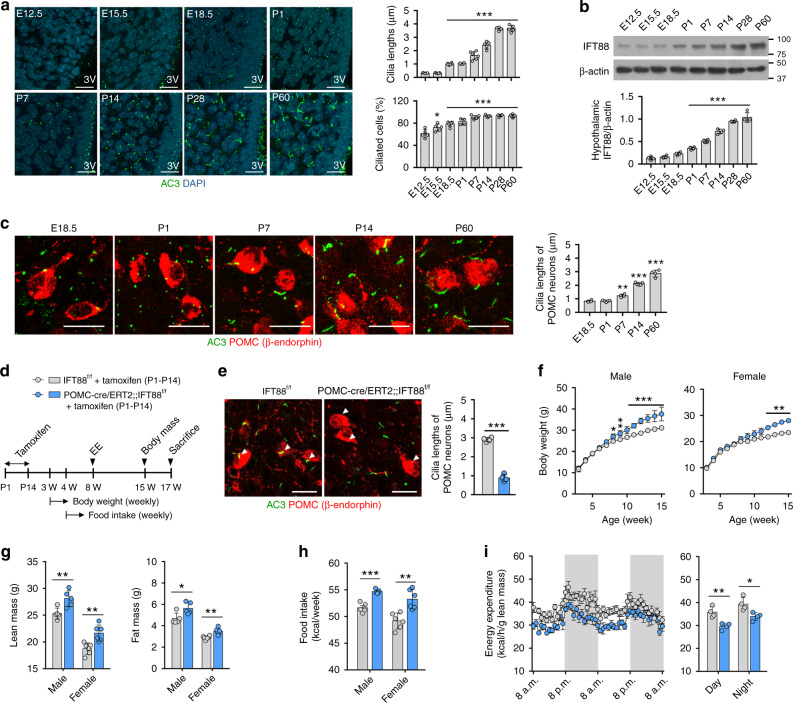
Ciliogenesis in POMC neurons during the early postnatal period is critical for adulthood energy balance. **a** Cilia (AC3) staining in the developing hypothalamus of C57 mice (*n* = 4 for P0 and P28, *n* = 5 for E12.5, E15.5, P14, and P60, *n* = 6 for E18.5 and P7). The graphs depict the average length of about 100 ARH cilia and the ciliated cell percentage (the cilia numbers divided by the DAPI numbers) in each mouse. Scale bars: 20 μm. 3V third cerebroventricle. **b** IFT88 immunoblotting in the developing hypothalamus of C57 mice (*n* = 4). **c** AC3 and β-endorphin double staining in the ARH of C57 mice showing POMC neuron ciliogenesis during development (*n* = 4 for E18.5, P7, and P60, *n* = 5 for P1 and P14). Scale bars: 20 μm. **d** Experimental scheme in mice with POMC-specific IFT88 depletion that was induced by tamoxifen injections during the postnatal period (P1–P14). **e** Representative cilia images showing ciliary dysgenesis in POMC neurons (*n* = 4). Arrowheads indicate cilia of POMC neurons. Scale bars: 20 μm. **f** Body weights in mice with postnatal ciliary dysgenesis in POMC neurons and their IFT88^f/f^ litters on a chow diet (*n* = 5 for males, *n* = 6 for females). **g** Lean mass and fat mass measured at 15 weeks (*n* = 5 for males, *n* = 6 for females). **h** Average values of weekly food intake during 10–15 weeks (*n* = 5 for males, *n* = 6 for females). **i** Energy expenditure measured at 12 weeks (*n* = 4). Data are presented as the mean ± SEM values. Statistics were performed using one-sided one-way ANOVA (**a**–**c**), one-sided two-way ANOVA (**f**) followed by post hoc LSD test and two-sided Student’s *t* test (**e**, **g**–**i**). *p < 0.05, **p < 0.01, ***p < 0.001 vs. IFT88^f/f^ controls or between the indicated groups.

We next asked when the cilia were formed during hypothalamic development. The very short cilia were observed in the hypothalamus of C57 mouse from E12.5 (Fig. 3a). The numbers and lengths of primary cilia gradually increased and these changes were prominent after birth (Fig. 3a). IFT88 immunoblotting also showed a gradual increase in hypothalamic IFT88 expression from E12.5 to postnatal day 60 (P60; Fig. 3b). POMC neuronal ciliogenesis showed a similar growth pattern to other hypothalamic cells (Fig. 3c).

A rapid ciliary elongation during the early postnatal period may imply the contribution of primary ciliogenesis to hypothalamic development in this period. To this end, we specifically inhibited POMC neuronal ciliogenesis during the postnatal period by injecting tamoxifen daily to POMC-cre/ERT2;;IFT88^f/f^ mice from P1 to P14 and subsequently monitored changes in energy metabolism (Fig. 3d). The cre-lox recombination was confirmed by the presence of short cilia and tdTomato expression in >90% of β-END^+^ POMC neurons at 7 weeks (Fig. 3e and Supplementary Fig. 1b). POMC-cre/ERT2;;IFT88^f/f^ mice receiving postnatal tamoxifen injections demonstrated a higher weight gain, which was significant after 8 weeks in males and 12 weeks in females (Fig. 3f). Moreover, these mice also showed an increased lean mass and fat mass at 15 weeks (Fig. 3g), although the degree of obesity was less than that observed in POMC-cre;;IFT88^f/f^ mice (Fig. 2c, f). Increased food intake and reduced EE was also observed in mice with early postnatal ciliary dysgenesis in POMC neurons (Fig. 3h, i). These data underscore the importance of postnatal ciliogenesis in POMC neurons for the maintenance of normal energy balance in adulthood. However, note that the obesity phenotype induced by early postnatal disruption of POMC ciliogenesis was less remarkable than that caused by POMC-specific disruption of ciliogenesis from the embryonic period. Therefore, ciliogenesis in POMC^+^ immature neurons during embryogenesis also affects adult body weights.

### Impaired neuronal circuit formation in cilia-defective developing POMC neurons

**Fig. 4:**
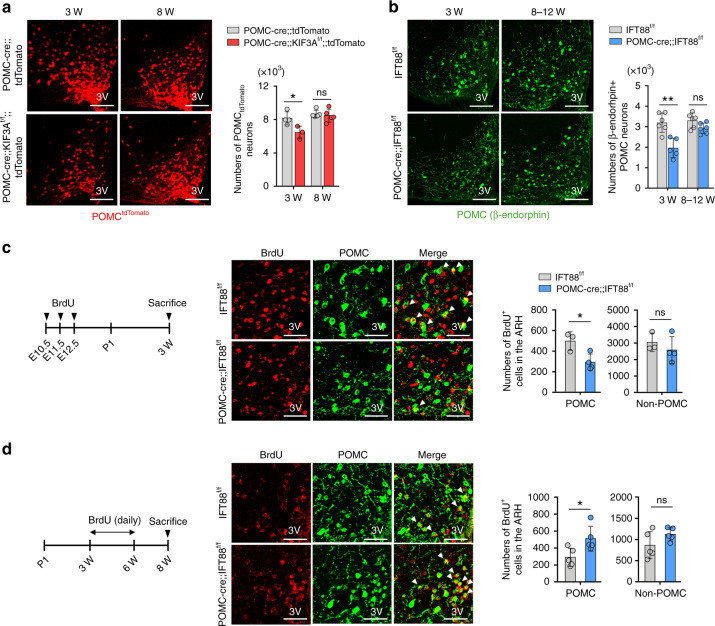
Altered neurogenesis in the developing POMC neurons with ciliary dysgenesis. **a** The numbers of tdTomato-labeled POMC neurons in the ARH of POMC-cre;;KIF3A^f/f^;;tdTomato mice compared with POMC-cre;;tdTomato littermates (*n* = 5 for POMC-cre;;IFT88^f/f^ mice at both ages, *n* = 4 for IFT88^f/f^ mice at 3 weeks, *n* = 3 for IFT88^f/f^ mice at 8 weeks). Measurements were conducted at the indicated ages. *p = 0.0367. Scale bars: 100 μm. 3V third cerebroventricle. **b** The numbers of β-endorphin-expressing POMC neurons in the ARH of POMC-cre;;IFT88^f/f^ mice and IFT88^f/f^ littermates at the indicated ages (*n* = 5 for POMC-cre;;IFT88^f/f^ mice and *n* = 6 for IFT88^f/f^ mice). **p = 0.002. Scale bars: 50 μm. **c** 5-Bromouridine (BrdU) and POMC (β-endorphin) double staining showing the reduced POMC neurogenesis during E10.5–E12.5 in POMC-cre;;IFT88^f/f^ mice (*n* = 4 for POMC-cre;;IFT88^f/f^ mice, *n* = 3 for IFT88^f/f^ mice). The experimental schedule was presented. Arrowheads indicate BrdU^+^ POMC neurons. *p = 0.023. Scale bars: 50 μm. **d** BrdU/β-endorphin double staining showing the increased neurogenesis of POMC neurons in POMC-cre;;IFT88^f/f^ mice during the post-weaning period (3–6 weeks) (*n* = 5). Arrowheads indicate BrdU^+^ POMC neurons. *p = 0.027. Scale bars: 50 μm. Data are presented as the mean ± SEM values. Statistics were performed using two-sided Student’s *t* test (**a**–**d**). ns not significant.

To gain mechanistic insights into the altered energy balance in POMC-specific cilia mutant mice, we examined changes in the POMC neuronal circuitry in cilia mutants. For this, we labeled POMC neurons with tdTomato through the mating of POMC-cre mice with tdTomato reporter mice. This labeling method enabled us to trace all POMC cell linages that transiently or permanently express POMC. The tdTomato^+^ POMC cell numbers were reduced by ~21% upon weaning but completely recovered to normal at 8 weeks in POMC-cre;;KIF3A^f/f^;;tdTomato mice (Fig. 4a). Similarly, the numbers of β-END^+^ terminally differentiated POMC neurons in POMC-cre;;IFT88^f/f^ mice were decreased by about ~23% at 3 weeks but showed no change at 8–12 weeks (Fig. 4b). Given the reduced POMC neuron number in early postnatal life, we assessed POMC neurogenesis during embryogenesis. To label newborn POMC cells, we injected daily 5-bromouridine (BrdU) from E10.5 to 12.5, when POMC neurogenesis is highly active^10^, and conducted BrdU/β-END double immunohistochemistry at 3 weeks. BrdU^+^ POMC cell numbers were decreased by approximately 30% in cilia mutants compared to those in the controls while BrdU^+^ non-POMC cell numbers remained unaltered (Fig. 4c). As POMC neuron numbers recovered to their normal levels at 8–12 weeks, we determined POMC neurogenesis during the post-weaning period. For this, we injected BrdU daily from 3 weeks to 6 weeks and sacrificed mice at 8 weeks. We observed a significant increase in BrdU^+^ POMC numbers in POMC-cre;;IFT88^f/f^ mice (Fig. 4d). Again, BrdU^+^ non-POMC cell numbers did not change. These findings suggested that a ciliary deficit impairs embryonic POMC neurogenesis, which may be rescued by cilia-independent neurogenesis during young adulthood. On the other hand, terminal deoxynucleotidyl transferase-mediated dUTP-fluorescein nick end labeling (TUNEL) staining performed at E16.5 and P14 showed no increase in POMC neuronal death (Supplementary Fig. 2), and thus ciliary defect does not promote the death of POMC neurons.

**Fig. 5:**
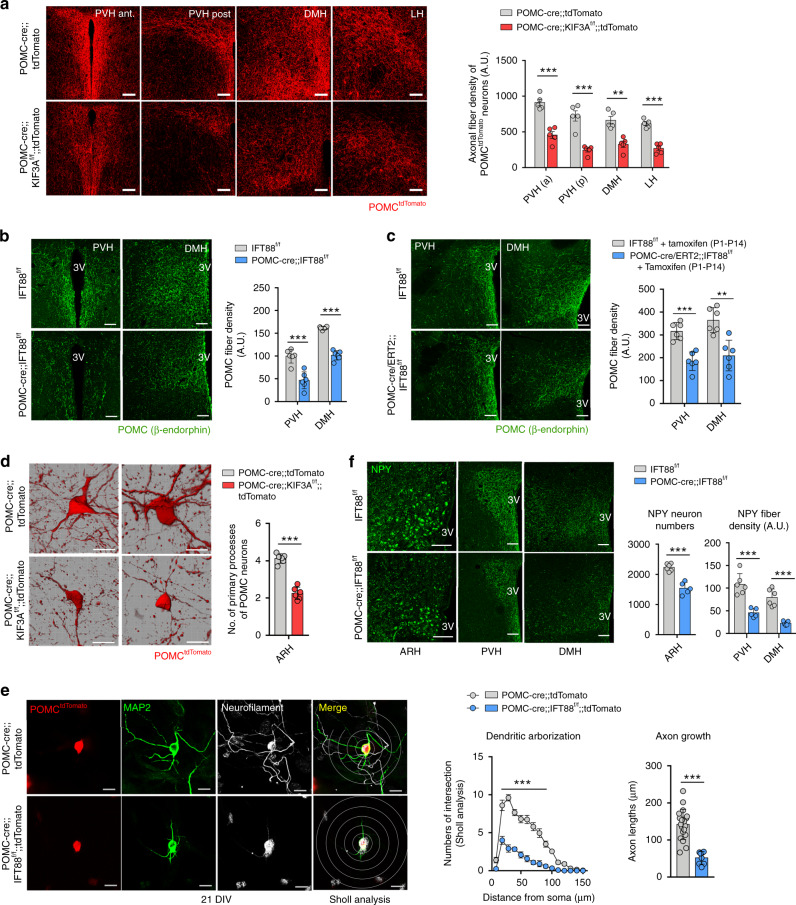
Impaired neuronal circuit formation in POMC neurons with defective cilia. **a** Axonal projections of tdTomato-labeled POMC neurons in hypothalamic paraventricular nucleus (PVH), dorsomedial nucleus (DMH) and lateral hypothalamic area (LH) of POMC-cre;;KIF3A^f/f^;; tdTomato and POMC-cre;;tdTomato male mice at 8 weeks (*n* = 5). Representative images and the graph depicting the axonal fiber density of POMC neurons in each hypothalamic area. Scale bars: 100 μm. **b**, **c** β-Endorphin-immunoreactive axonal projections of POMC neurons in the PVH and DMH of POMC-cre;;IFT88^f/f^ and POMC-cre/ERT2;;IFT88^f/f^ mice and their control littermates at 8–10 weeks (*n* = 6). Scale bars: 100 μm. **d** The primary neuronal process of tdTomato^+^ POMC neurons in POMC-cre;;KIF3A^f/f^;;tdTomato and POMC-cre;;tdTomato mice (*n* = 6). Representative images and graph depicting the average number of the primary process of 100 POMC neurons. Scale bars: 20 μm. ARH hypothalamic arcuate nucleus. **e** MAP2 (dendrite marker), neurofilament (axon marker), and tdTomato (POMC neuron) triple immunostaining in primary cultured hypothalamic neurons at 21 days in vitro (DIV), which were obtained from POMC-cre;;IFT88^f/f^;;tdTomato and POMC-cre;;tdTomato embryos, respectively. Sholl analysis was conducted for the assessment of dendritic arborization in 20 POMC neurons from POMC-cre;;tdTomato embryos and 12 neurons from POMC-cre;;IFT88^f/f^;;tdTomato embryos. The full lengths of axons were measured for 19 cells from POMC-cre;;tdTomato embryos and 8 cells from POMC-cre;;IFT88^f/f^;;tdTomato embryos. Scale bars: 20 μm. **f** NPY immunostaining in the ARH, PVH, and DMH of 3-week-old POMC-cre;;IFT88^f/f^ mice and IFT88^f/f^ mice (*n* = 6 for IFT88^f/f^ mice, *n* = 5 for POMC-cre;;IFT88^f/f^ mice). Scale bars: 100 μm. Data values are presented as mean ± SEM. Statistics were performed using two-sided Student’s *t* test (**a**–**d**, **e**—axon, **f**) and one-sided two-way ANOVA followed by post hoc LSD test (**e**—dendrite). **p < 0.01, ***p < 0.001 between the indicated groups.

We next examined axonal projections of POMC neurons with ciliary dysgenesis. In 8-week-old POMC-cre;;KIF3A^f/f^;;tdTomato mice, tdTomato^+^ POMC fiber density was profoundly reduced in the hypothalamic paraventricular (PVH), dorsomedial (DMH), and lateral hypothalamic (LH) areas (Fig. 5a). Likewise, β-END-immunoreactive fiber density in the PVH and DMN was decreased in POMC-cre;;IFT88^f/f^ mice as well as in POMC-cre/ERT2;;IFT88^f/f^ mice with early postnatal tamoxifen injection (Fig. 5b, c). In addition, the numbers of primary processes of tdTomato^+^ POMC neurons was also reduced in cilia-defective POMC neurons (Fig. 5d).

To differentiate the effect of primary cilia on axon and dendrite growth, we cultured tdTomato^+^ POMC neurons derived from POMC-cre;;tdTomato embryos and POMC-cre;;IFT88^f/f^;;tdTomato embryos for up to 21 days and performed triple staining of tdTomato, neurofilament (axon marker), and MAP2 (dendrite marker). IFT88-deleted POMC neurons exhibited a marked reduction in both dendrite branching and axon lengths after 14 and 21 days in vitro culture (Fig. 5e and Supplementary Fig. 3), suggesting impaired axon and dendrite outgrowth in POMC neurons with disrupted ciliogenesis. A certain proportion of ARH NPY/AGRP neurons originate from the POMC-expressing neuronal precursors^10^. Indeed, NPY immunostaining revealed a significant decrease in the NPY neuron number and axonal fiber density in POMC-cre;;IFT88^f/f^ mice at 3 weeks (Fig. 5f). All these evidence indicated that ciliary defects disrupt normal circuit formation in the neurons derived from embryonic POMC-expressing neural progenitors.

### Lysosomal dysfunction in the POMC neurons with defective ciliogenesis

**Fig. 6:**
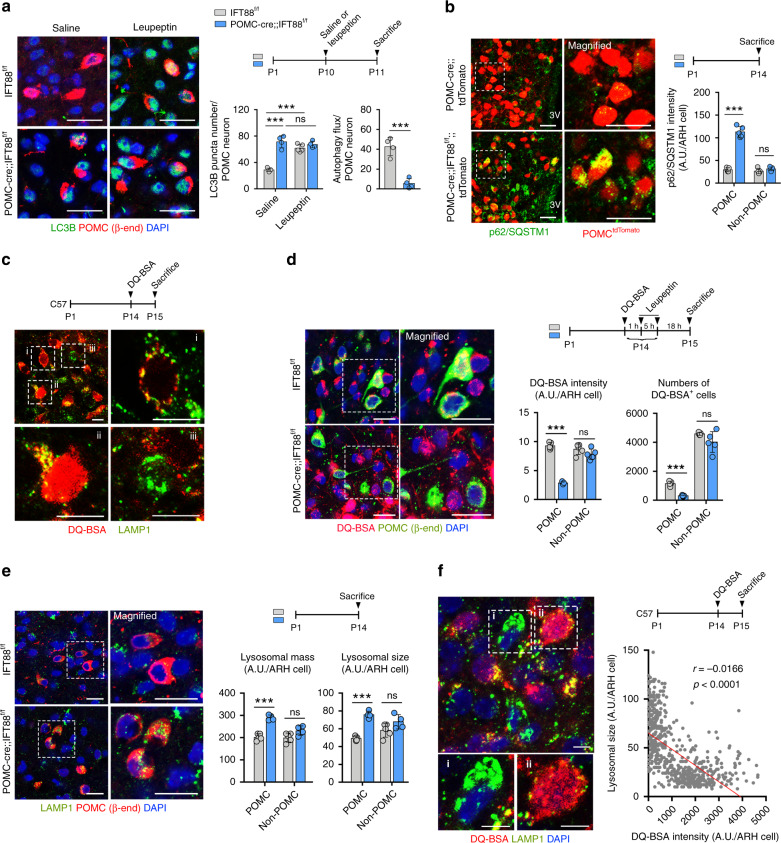
Lysosomal dysfunction in the POMC neurons with defective ciliogenesis. **a** LC3B (autophagy marker), β-endorphin (β-END), and DAPI staining for autophagy analysis in the ARH of POMC-cre;;IFT88^f/f^ and IFT88^f/f^ neonates at P11 (*n* = 5 for saline, *n* = 4 for leupeptin each genotype). Scale bars: 20 μm. **b** p62 (autophagy substrate) and tdTomato costaining in the ARH of POMC-cre;;IFT88^f/f^;;tdTomato and POMC-cre;;tdTomato neonates at P14 (*n* = 5). The graph depicts the average fluorescence intensity of p62 in 100 cells per mouse. Scale bars: 20 μm. **c** Double staining of DQ-BSA, indicative of lysosomal protein degradation, and LAMP1 in the ARH of C57 mice (*n* = 6). Magnified images of (i) ARH cell with DQ-BSA signals merged with lysosomes, (ii) cell with high-intensity lysosomal and extralysosomal DQ-BSA signals, and (iii) cell with lack of DQ-BSA signals. Scale bars: 10 μm. **d** Double staining of DQ-BSA and β-END in the ARH of POMC-cre;;IFT88^f/f^ mice and IFT88^f/f^ littermates (*n* = 5). The average DQ-BSA intensity values in 100 cells and the numbers of DQ-BSA^+^ cells in the ARH are presented. Scale bars: 20 μm. **e** LAMP1 and β-END double staining showing lysosomal mass and sizes in POMC and non-POMC ARH cells at P14 (*n* = 4 for POMC-cre;;IFT88^f/f^ mice, *n* = 5 for IFT88^f/f^ mice). The graph depicts the average values of LAMP1 intensity (lysosomal mass) and LAMP1^+^ puncta size (lysosomal size) in 100 cells per mouse. Scale bars: 20 μm. **f** Double immunostaining of DQ-BSA and LAMP1 in the ARH cells of C57 mice (*n* = 6). Magnified images of (i) ARH cell with larger lysosomes and lower DQ-BSA fluorescence intensity and (ii) cell with smaller lysosomes and higher DQ-BSA intensity. The graph depicts the correlation between the average lysosomal size and DQ-BSA intensity in ARH cells. Scale bar: 5 μm. Data values are presented as mean ± SEM. Statistics were performed using two-sided Student’s *t* test (**a**—autophagy flux, **b**, **d**, **e**), one-sided one-way ANOVA followed by post hoc LSD test (**a**—LC3 puncta), and linear regression analysis (**f**). ***p < 0.001 between groups. ns not significant.

We further explored the mechanism by which defective ciliogenesis disrupts axonal projections in POMC neurons. Autophagy is a catabolic process that degrades organelles, long-lived proteins, and lipids to maintain cellular homeostasis^34^. An intimate crosstalk between primary cilia and the autophagic machinery has been reported^35^. Moreover, autophagy defect in developing POMC neurons reduces axon growth and induces adiposity and glucose intolerance^36^. We therefore examined the autophagic flux in POMC neurons by measuring light chain 3B (LC3B) expression under the conditions with or without administration of the lysosomal protease inhibitor leupeptin. This analysis was conducted at P10–P11 as formation of POMC neuronal circuits actively takes place in the second postnatal week^11^. In IFT88-depleted POMC neurons, LC3B puncta numbers in saline-injected condition was markedly elevated but the leupeptin-induced increase in LC3B puncta numbers, a measure of autophagic flux, was profoundly reduced (Fig. 6a). Consistently, the expression of autophagy substrate p62/SQSTM1 was elevated in POMC neurons depleting IFT88 (Fig. 6b), indicating a reduced lysosomal degradation of autophagic substrates.

We also assessed the capacity for autophagy-independent lysosomal protein degradation by using DQ-BSA, which produces red fluorescent products upon hydrolysis by lysosomal proteases. We first examined lysosomal protein degradation by using DQ-BSA in the hypothalamus of normal mice. For this, DQ-BSA was injected into the third cerebroventricle of C57 mice at P14 and then examined the DQ-BSA fluorescent intensity in the ARH 24 h later. The DQ-BSA fluorescence was coexpressed with lysosomal-associated membrane protein 1 (LAMP1)-expressing late endosome and lysosomes in some hypothalamic cells (Fig. 6ci). These data suggested that lysosomal hydrolysis of DQ-BSA takes place in the mouse hypothalamus. DQ-BSA fluorescence was also observed in extralysosomal compartments especially in cells with strong DQ-BSA signals (Fig. 6cii) and thus DQ-BSA might exit the lysosomes after degradation and traffic to the extralysosomal compartments. Some cells were lacking DQ-BSA signals (Fig. 6ciii). This could be due to no lysosomal hydrolysis or complete degradation of DQ-BSA.

We next examined the lysosomal protein degradation in POMC-cre;;IFT88^f/f^ mice and their wild littermates at P14. To reduce lack of DQ-BSA signals due to excessive lysosomal degradation, we injected leupeptin 1 h and 6 h after DQ-BSA administration. Importantly, DQ-BSA fluorescence was robustly reduced in POMC neurons in POMC-cre;;IFT88^f/f^ mice (Fig. 6d). Moreover, the number of DQ-BSA^+^ POMC cells was also reduced (Fig. 6d). In contrast, no alteration was observed in the DQ-BSA fluorescence intensity of non-POMC cells (Fig. 6d). This finding revealed a regulatory role for primary cilia in the lysosomal protein degradation capacity. LAMP1 staining further showed an increase in the lysosomal mass and sizes in POMC neurons lacking IFT88 but no lysosomal changes in non-POMC cells at P14 (Fig. 6e), which may be a compensatory change to the reduced lysosomal hydrolysis capacity. Consistent with this, DQ-BSA/LAMP1 costaining in the hypothalamus of C57 mice demonstrated that cells with larger lysosomes exhibited lower DQ-BSA fluorescence intensity (Fig. 6fi). In contrast, cells with smaller lysosomes had a high DQ-BSA signal (Fig. 6fii).

### Interplay among cilia, lysosomal protein degradation, and axonal projection in the hypothalamic neurons

**Fig. 7:**
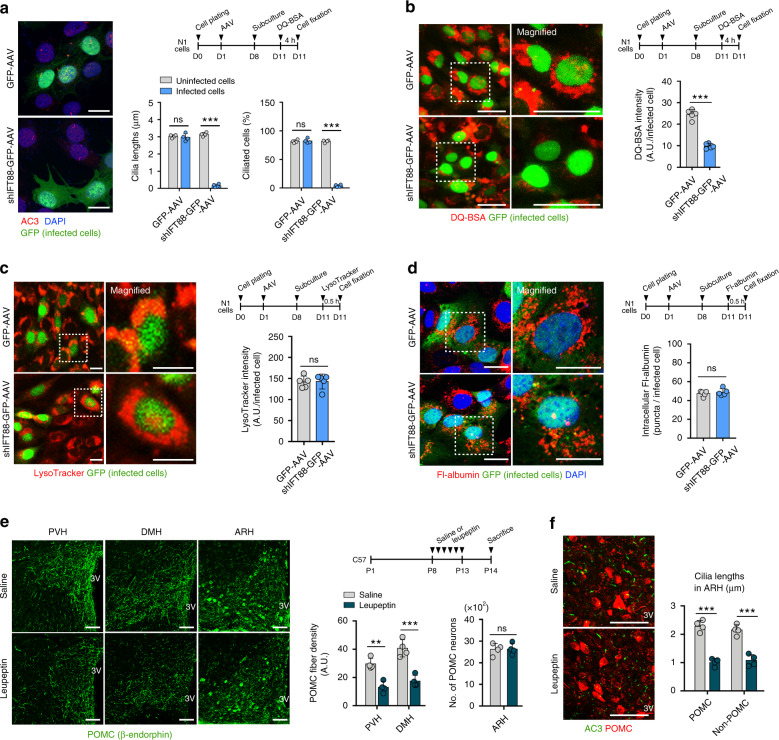
Interplay among cilia, lysosomal protein degradation, and axonal projection in the hypothalamic neurons. **a** Confirmation of ciliary dysgenesis in N1 hypothalamic neuron cells expressing IFT88 small hairpin RNA (shIFT88)-GFP. ARL13B (cilia marker), GFP, and DAPI staining in N1 cells transfected with shIFT88-GFP-expressing AAV (adeno-associated virus) or GFP-AAV (*n* = 4 wells). The average cilia lengths and ciliated cell percentages of about 50 infected cells and 50 uninfected cells are presented in each treatment group. Scale bars: 20 μm. **b** DQ-BSA intensity in N1 hypothalamic neuron cells infected with either shIFT88-GFP-AAV or GFP-AAV (*n* = 5 wells). The average DQ-BSA intensity of 50 GFP^+^ cells per well was presented. Scale bars: 20 μm. **c** Immunostaining of lysosomes using LysoTracker dye in N1 hypothalamic neuron cells (*n* = 5 wells). The average values of about 50 infected cells per well are presented. Scale bar: 10 μm. **d** Fluorescent-labeled albumin (Fl-albumin), GFP, and DAPI staining in N1 hypothalamic neuron cells transfected with shIFT88-GFP-AAV or GFP-AAV (*n* = 5 wells). The average numbers of Fl-albumin^+^ puncta in 50 GFP-expressing cells were measured per well. Scale bars: 20 μm. **e** β-END immunostaining in the hypothalamus of C57 neonates injected with saline or leupeptin during P8–P13 (*n* = 4). Scale bars: 100 μm. **f** POMC (β-END) and cilia (AC3) double immunostaining in neonates with saline or leupeptin injections (*n* = 4). Scale bars: 100 μm. Data are presented as mean ± SEM. Statistics were performed using two-sided Student’s *t* test (**a**–**f**). **p < 0.01 and ***p < 0.001 between groups. ns not significant.

To further clarify the observed linkage between lysosomal changes and ciliary defect, we examined whether the inhibition of ciliogenesis in N1 hypothalamic neuronal cells caused lysosomal changes. N1 hypothalamic neuron cells are highly ciliated and have been previously used for the study of hypothalamic neuron cilia^14^. Cells were transfected with an adeno-associated virus (AAV) expressing IFT88 short hairpin RNA (shIFT88)-green fluorescent protein (GFP) or GFP alone and then maintained under the low (1%) serum condition for 48 h before staining. Expression of shIFT88-GFP successfully inhibited ciliogenesis in infected cells but the expression of GFP alone did not affect ciliogenesis (Fig. 7a). N1 cells expressing shIFT88-GFP showed a dramatic reduction in the DQ-BSA fluorescent intensity (Fig. 7b) but no alteration in the LysoTracker fluorescence intensity, a measure of lysosomal mass (Fig. 7c). Cellular uptake of fluorescence-labeled albumin was unaltered in shIFT88-GFP-expressing cells (Fig. 7d), and thus reduced DQ-BSA intensity may not be due to the decreased uptake of DQ-BSA. These findings strongly suggested that ciliogenesis is linked to cellular lysosomal protein degradation capacity.

We further tested whether the inhibition of lysosomal proteolysis could disrupt axonal projections from POMC neurons. Indeed, an intraperitoneal injection of leupeptin from P8 to P13 in C57 neonates significantly decreased the POMC axonal fiber intensity in the PVH and DMN without alterations in the number of POMC neurons (Fig. 7e). These findings demonstrated that lysosomal protein degradation is required for POMC neuron circuit organization. In addition, leupeptin treatment significantly repressed hypothalamic ciliogenesis (Fig. 7f). Therefore, lysosomal protein degradation may be also required for cilia formation in the developing hypothalamic neurons.

### The neonatal leptin surge-stimulated hypothalamic ciliogenesis is critical for POMC neuronal circuit formation

**Fig. 8:**
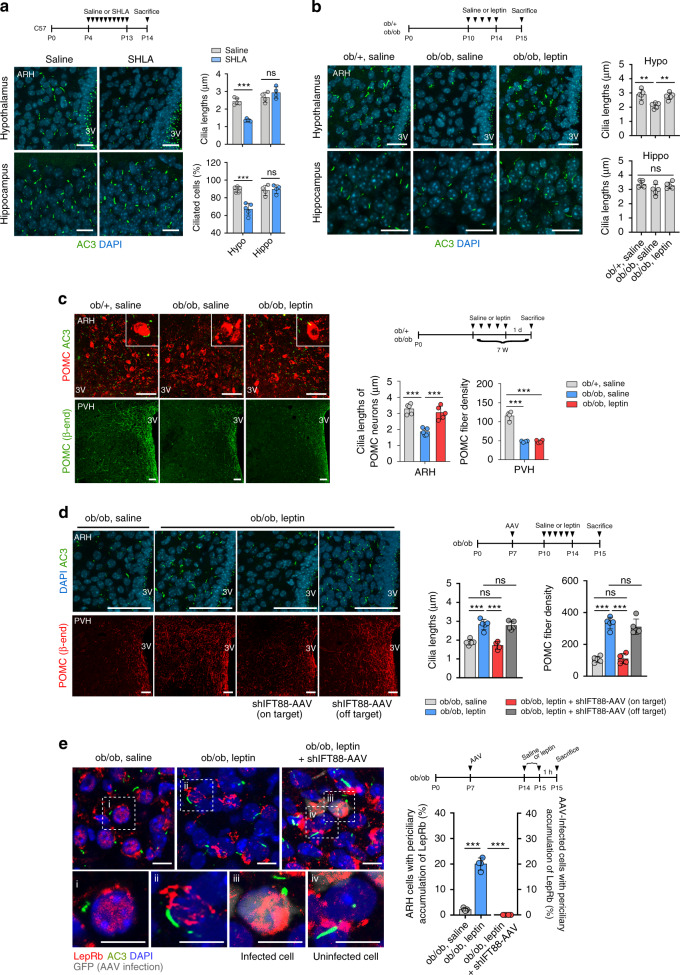
The neonatal leptin surge stimulates ciliogenesis and neural circuit formation in the hypothalamus. **a** Cilia (AC3) staining in the hypothalamic ARH and hippocampal dentate gyrus of C57 neonates at P14 that received either saline or leptin antagonist SHLA injections from P4 to P13. The average lengths of 100 cilia and the ciliated cell percentage in each area per mouse are presented (*n* = 5 for hypothalamus, *n* = 4 for hippocampus). Scale bars: 20 μm. **b** Cilia staining in the hypothalamus (Hypo) and hippocampus (Hippo) of ob/+ mice, ob/ob mice, and ob/ob mice with leptin treatment (P10–P14) (*n* = 5). The average lengths of 100 cilia in each area per mouse are presented. Scale bars: 20 μm. **c** Upper panel: cilia (AC3) and POMC (β-END) costaining in the hypothalamic ARH in 7-week-old ob/+ mice, ob/ob mice, and ob/ob mice with adulthood leptin replacement (10 mg/kg/day for 7 days; *n* = 5). Lower panel: axonal projections of POMC neurons in the three groups (*n* = 4). Scale bars: 50 μm. **d** Ciliogenesis in the ARH and POMC axonal projection to the PVH in ob/ob neonates injected with saline, leptin alone, or leptin + shIFT88-GFP-AAV (*n* = 4 for AAV on-target group, *n* = 5 for the other 3 groups). The mice with off-target AAV injection were used as a control. Scale bars: 50 μm. **e** AC3 (cilia), functional leptin receptor (LepRb), GFP (AAV transfection), and DAPI costaining in the ARH of ob/ob neonates injected with saline, leptin, or leptin + shIFT88-GFP-AAV. The percentages of ARH cells and AAV-infected cells with periciliary leptin receptor accumulation were counted (*n* = 5 for the ob/ob-saline group and *n* = 4 for the other 2 groups). Scale bars: 10 μm. Data values are presented as mean ± SEM. Statistics were performed using two-sided Student’s *t* test (**a**) and one-sided one-way ANOVA (**b**–**e**) followed by post hoc LSD test. **p < 0.01, ***p < 0.001 between the indicated groups. ns not significant.

Adipocyte-derived leptin is known as a critical hormonal cue for hypothalamic development^12^. Supporting this role, the plasma leptin levels increase during the second postnatal week independent of fat mass and decline after weaning^37^. During this period, leptin triggers axonal outgrowth of hypothalamic neurons to establish neuronal circuitry required for normal body weights^12^. To test whether this postnatal leptin surge may control hypothalamic ciliogenesis, the leptin antagonist SHLA was injected intraperitoneally to C57 neonates from P4 to P13. The blockade of leptin actions during this period profoundly repressed hypothalamic ciliogenesis (Fig. 8a). It was noteworthy that SHLA treatment did not alter ciliogenesis in multi-ciliated ependymal cells surrounding the third ventricle and in other brain areas, such as the hippocampus (Fig. 8a). Consistently, leptin-deficient ob/ob neonates had reduced hypothalamic ciliogenesis compared to ob/+ littermates, an effect which was rescued by leptin replacement (10 mg/kg/day) from P10 to P14 (Fig. 8b). Again, these leptin-related ciliary changes were not observed in the hippocampus (Fig. 8b). Thus leptin regulates hypothalamic ciliogenesis in the early postnatal period in a brain region-specific manner. Notably, however, replacement of leptin in 7-week-old adult ob/ob mice recovered the reduction in hypothalamic ciliogenesis but not in POMC axonal projections (Fig. 8c). Thus leptin regulates hypothalamic ciliogenesis in both neonatal and adult mice. In contrast, leptin regulation of POMC neural circuit formation may occur in a restricted period, i.e., early postnatal life in mice.

Considering the overlapping role of leptin and the primary cilia in regulating POMC neuronal wiring, we postulated that primary cilia may mediate leptin actions. To this end, we inhibited hypothalamic ciliogenesis by microinjecting shIFT88-GFP-AAV into the ARH of ob/ob neonates at P7 and then administered leptin intraperitoneally during P10–P14. The hypothalamus was collected at P15. Successful ARH expression of shIFT88-GFP blocked leptin-induced ciliogenesis and POMC axonal projections in ob/ob neonates but did not change the POMC neuron numbers (Fig. 8d and Supplementary Fig. 4). An off-target AAV injection could not block the effects of leptin (Fig. 8d and Supplementary Fig. 5), and thus we used off-target injections as a control. These findings support that the cilia mediate the leptin regulation of hypothalamic neuronal circuit formation.

It has been shown that the functional leptin receptor LepRb is translocated to the periciliary region in response to leptin treatment in hypothalamic neurons^38^. Consistently, we found that leptin administration in ob/ob neonates caused periciliary accumulation of LepRb in hypothalamic neurons (Fig. 8e). This response was significantly blunted in shIFT88-infected ARH neurons (Fig. 8e). Therefore, primary cilia may mediate leptin receptor trafficking to the periciliary area upon treatment with leptin.

### Leptin triggers lysosomal proteolysis in hypothalamic neurons in cilia-dependent mechanism

**Fig. 9:**
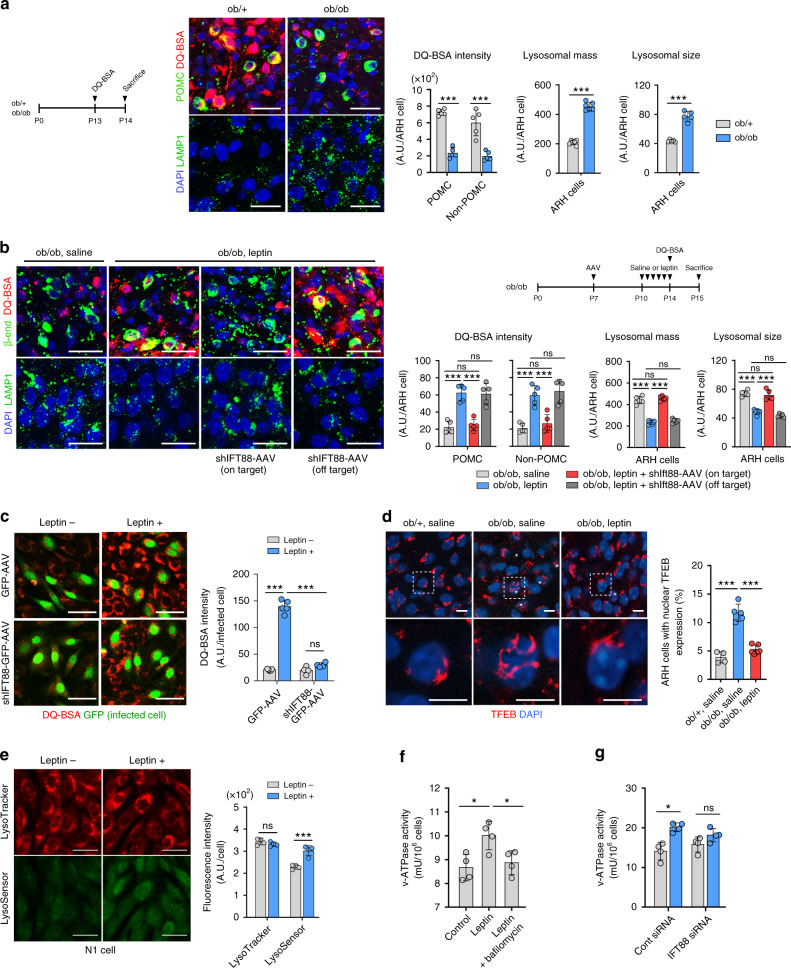
Leptin stimulates lysosomal protein degradation in the hypothalamic neurons via cilia-dependent mechanism. **a** Representative images of DQ-BSA/β-END staining and LAMP1/DAPI staining in the hypothalamic ARH of ob/+ and ob/ob mice at P14 (*n* = 5). The graphs depict the average values of DQ-BSA fluorescence intensity (lysosomal protein degradation), LAMP1 intensity (lysosomal mass) and LAMP1^+^ puncta size (lysosomal size) in 100 ARH cells per mouse. Scale bars: 50 μm. 3V third ventricle. **b** DQ-BSA/β-END and LAMP1/DAPI staining in the ARH of ob/ob mice injected with saline, leptin alone, or leptin + shIFT88-GFP-AAV (*n* = 5). The average values of DQ-BSA and LAMP1 intensity and puncta size in 100 ARH cells per mice are presented. Scale bars: 50 μm. **c** DQ-BSA fluorescence intensity in N1 cells transfected with GFP-AAV or shIFT88-GFP-AAV and treated with leptin (100 nM for 1 h) (*n* = 4 wells for GFP-AAV, *n* = 5 wells for shIFT88-GFP-AAV). The DQ-BSA fluorescence intensity of 50 GFP^+^ cells per well was analysed. Scale bars: 50 μm. **d** TFEB/DAPI double staining, as a measure of lysosomal biogenesis, in the ARH of ob/+ mice with saline and ob/ob mice with saline or leptin treatment (*n* = 4 for ob/+ saline, *n* = 5 for ob/ob saline, and *n* = 6 for ob/ob leptin). Asterisks indicate cells with nuclear TFEB expression. Scale bars: 10 μm. **e** Lysosomal staining using LysoTracker and pH-sensitive LysoSensor dyes in N1 cells with or without leptin treatment (*n* = 4 wells). The average fluorescent intensity of 100 cells per well was analysed. Scale bars: 50 μm. **f** The vacuolar H^+^-ATPase (v-ATPase) activity in N1 cells treated with leptin alone or leptin with lysosomal v-ATPase inhibitor bafilomycin (*n* = 4 wells). **g** The v-ATPase activity in N1 cells transfected with either control small inhibitory RNA (siRNA) or IFT88 siRNA and then treated with leptin (*n* = 4 wells). Data values are presented as mean ± SEM. Statistics were performed using two-sided Student’s *t* test (**a**, **e**, **g**) and one-sided one-way ANOVA (**b**–**d**, **f**) followed by post hoc LSD test. *p < 0.05 and ***p < 0.001 between the indicated groups. ns not significant.

We further tested whether leptin regulates lysosomal function in the developing hypothalamus. We observed a remarkable decrease in the DQ-BSA fluorescence intensity in the ARH POMC neurons and non-POMC cells in ob/ob mice (Fig. 9a). This reduction was completely rescued by leptin treatment during the postnatal second week (Fig. 9b). Likewise, leptin treatment in adult ob/ob mice also increased hypothalamic DQ-BSA fluorescence intensity (Supplementary Fig. 6). Disruption of ciliogenesis prevented leptin-promoted lysosomal protein degradation in vivo and in vitro (Fig. 9b, c). In contrast, the lysosomal sizes, an indicator of lysosomal dysfunction, and the lysosomal mass were increased in the ARH neurons of ob/ob neonates and decreased by leptin treatment (Fig. 9a, b). Simultaneously, we assessed lysosomal biogenesis by examining the nuclear translocation of TFEB, a master transcriptional factor of lysosomal biogenesis^39^. The number of hypothalamic neurons with nuclear TFEB expression was significantly increased in ob/ob mice, which was decreased upon treatment with leptin (Fig. 9d). In hypothalamic N1 cells, leptin did not alter the LysoTracker intensity (Fig. 9e). These findings suggested that leptin may not directly control the lysosomal mass. Thus increased lysosomal biogenesis in the hypothalamus of ob/ob mice might occur to compensate for reduced lysosomal protein degradation.

We further investigated the mechanism by which leptin regulates lysosomal protein degradation. In hypothalamic N1 cells, leptin treatment significantly increased the LysoSensor intensity (Fig. 9e) and thus lowered lysosomal pH. As lysosomal proteases are more active at lower pH^40^, leptin may stimulate lysosomal protease activity by facilitating lysosomal acidification processes. Lysosomal vacuolar ATPase (v-ATPase) is a proton pump responsible for lysosomal acidification^41^. The v-ATPase activity was significantly increased following leptin treatment and this effect was completely blocked by the lysosomal v-ATPase inhibitor bafilomycin and by an IFT88 knockdown (Fig. 9f, g). These data suggested that leptin may increase bafilomycin-sensitive lysosomal v-ATPase activity through cilia-dependent mechanisms. Overall, these findings indicate that leptin acts on ARH neurons to stimulate lysosomal protein degradation via a cilia-dependent regulation of lysosomal acidification.

### Maternal malnutrition disrupts the leptin regulation of hypothalamic cilia–lysosome–neuronal circuit formation in offspring

**Fig. 10:**
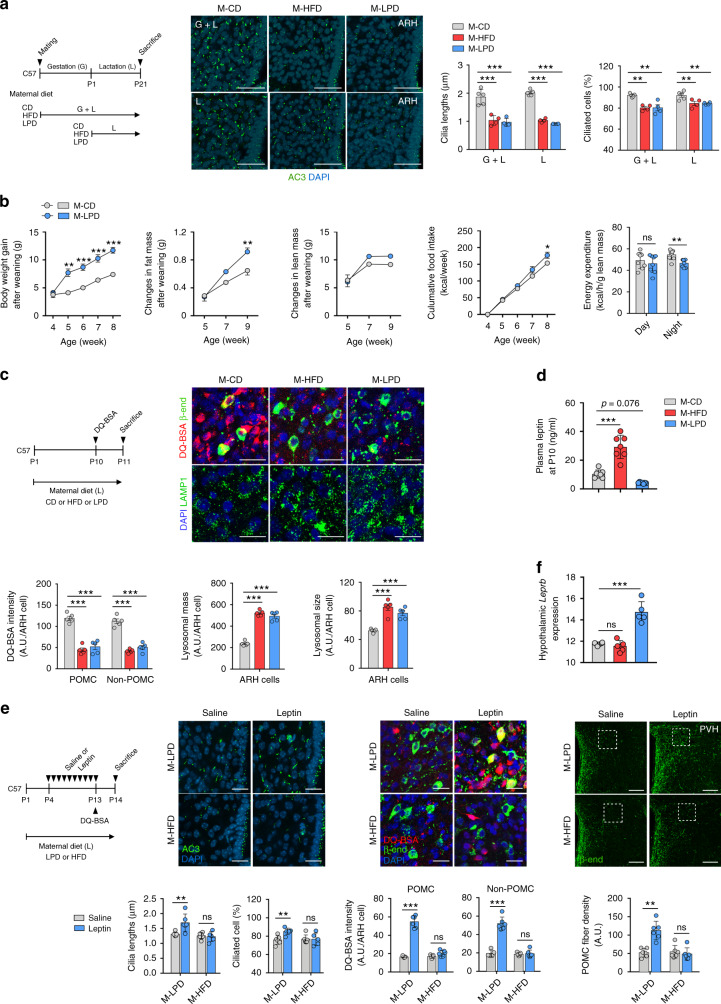
Maternal malnutrition disrupts the leptin regulation of hypothalamic cilia–lysosome–neuronal circuit formation in neonatal mice. **a** Hypothalamic cilia images in the neonatal offspring (at P14) of dams fed a high-fat diet (HFD) or low protein diet (LPD) during gestation and lactation or during lactation (*n* = 5 for M-CD, *n* = 4 for M-HFD and M-LPD). The average lengths of 100 ARH cilia per mouse are presented. Scale bars: 50 μm. **b** Changes in body weights, fat and lean mass, cumulated food intake, and energy expenditure (at 4 weeks) during the post-weaning period in the offspring of CD- or LPD-fed dams (*n* = 4 for food intake monitoring, *n* = 8 for other measurement). **c** DQ-BSA/β-END and LAMP1/DAPI double staining in the hypothalamic ARH of offspring of dams on a HFD or LPD (*n* = 5). The average values of 100 ARH cells per mouse are presented. Scale bars: 50 μm. **d** Plasma leptin concentrations in the offspring of CD-, HFD-, or LPD-fed dams during lactation at P10 (*n* = 7). **e** Hypothalamic cilia staining (AC3), lysosomal protein degradation (DQ-BSA/β-END staining) in the ARH, and POMC axonal projection in the PVH in the pups nourished by dams on a HFD or LPD and received either saline or leptin injections during P4–P13 (*n* = 5 for DQ-BSA study and *n* = 6 for all other analyses). Scale bars: 20 and 100 μm (axonal projections). **f** Hypothalamic leptin receptor (*Leprb*) mRNA expression in the offspring of CD-, LPD-, or HFD-fed dams at P14 (*n* = 6 for M-CD, *n* = 5 for the other 2 groups). Data values are presented as mean ± SEM. Statistics were performed using one-sided one-way ANOVA (**a**, **c**, **d**, **f**) and one-sided two-way ANOVA (**b**) followed by post hoc LSD test and two-sided Student’s *t* test (**b**—energy expenditure, **e**). *p < 0.05, **p < 0.01, and ***p < 0.001 between the indicated groups. ns not significant.

Maternal overnutrition and undernutrition have been reported to disrupt hypothalamic neuronal circuit organization and thereby implicate adulthood energy metabolism^42^. Thus we tested whether a maternal HFD and low protein diet (LPD) may affect hypothalamic ciliogenesis in offspring during the early postnatal period. In the experiment, dams were fed a standard chow diet (CD), 60% HFD, or 6% LPD from mating to weaning or upon delivery to weaning. A maternal HFD and LPD throughout pregnancy and lactation profoundly decreased the cilia length and frequency in the ARH of neonates (Fig. 10a). Notably, maternal overnutrition and undernutrition during breastfeeding period only induced comparable ciliary changes in the hypothalamic ARH (Fig. 10a). These ciliary changes were not observed in the cortex (Supplementary Fig. 7). As previously reported^43,44^, maternal HFD and LPD disrupted the development of POMC neuronal circuits without the alteration of the POMC neuron numbers (Supplementary Fig. 8), which may lead to altered energy metabolism. Indeed, pups nourished by LPD-fed dams showed an accelerated weight gain after weaning and greater increase in fat mass until around 10 weeks, which may be due to higher food intake and lower EE relative to their body sizes (Fig. 10b). These data indicated the considerable influence of the maternal diet during the lactation period on hypothalamic ciliogenesis, neuronal circuit formation, and metabolic alterations in later life.

We also tested whether maternal diets during breastfeeding alter the lysosomal function of developing ARH neurons. The DQ-BSA intensity was profoundly decreased in the ARH POMC and non-POMC cells of the offspring of HFD- and LPD-fed dams compared with those from CD-fed dams (Fig. 10c). These data resembled the reduction in the lysosomal proteolysis capacity observed in neonatal ob/ob mice. Moreover, LAMP1 staining revealed the increased lysosomal mass and sizes in the hypothalamus of the offspring of HFD- and LPD-fed dams (Fig. 10c).

Previous studies indicated that postnatal leptin levels were affected by the maternal diets^44,45^. In our analysis, the plasma leptin levels measured at P10 were found to be increased by maternal HFD and decreased by maternal LPD, implying leptin-resistant and leptin-deficient state, respectively (Fig. 10d). We finally tested whether postnatal leptin treatment could rescue a maternal poor nutrition-induced impairment in hypothalamic ciliogenesis, lysosomal protein degradation, and neural circuit formation. Leptin injections during P4–P13 stimulated hypothalamic ciliogenesis, lysosomal protein degradation, and POMC axonal projections in the offspring of LPD-fed mothers but not in those from HFD-fed mothers (Fig. 10e). The hypothalamic functional leptin receptor expression was elevated in the offspring of LPD-fed dams but unaltered in those of HFD-fed dams (Fig. 10f), which may account for the difference in the leptin responsiveness between two groups.

## Discussion

In our work, ciliary dysgenesis in adult POMC neurons, by tamoxifen-induced deletion of IFT88 at 7 weeks, had no significant effects on energy balance. As successful cre-lox recombination was observed in >90% of adult POMC neurons in this mouse model, these findings suggest a non-essential role of primary cilia of adult POMC neurons in the maintenance of energy balance. By contrast, primary cilia in MC4R-expressing POMC target neurons play a critical role in normal energy balance in the adulthood^28^. MC4R is colocalized with AC3 in the primary cilia. Moreover, human obesity-associated MC4R mutations disrupt the ciliary localization of MC4R, implying the importance of ciliary localization of MC4R in the maintenance of normal energy balance.

During embryogenesis, AC3-positive very short cilia were observed in the hypothalamic ventricular zone at E12.5. A blockade of ciliogenesis in POMC-expressing cells from the mid-gestation led to severe obesity, indicating an importance of embryonic ciliogenesis of these cells for adulthood energy balance. On the other hand, cilia elongation actively occurred in the early postnatal period when hypothalamic neuron maturation and circuit formation take place. Notably, inhibition of POMC ciliogenesis restricted to this postnatal period (P1–P14) significantly increased body weights and fat mass. Therefore, POMC neuronal ciliogenesis in the early postnatal period also affects adulthood adiposity.

The primary cilia regulate the self-renewal capacity of neuronal stem cells by relaying Shh signaling^20^. In line with this, POMC-cre;;IFT88^f/f^ mice showed the reduced POMC neurogenesis during E10.5–12.5 and about 20% reduction in POMC neuron numbers upon weaning. Interestingly, this reduction recovered to normal by 8–12 weeks. Therefore, adult neurogenesis may compensate for the reduction in the embryonic neurogenesis of POMC neurons. Notably, however, the recovery of the POMC neuron number in our cilia mutants in adulthood neither rescued the reduced axonal fiber density nor prevented the development of obesity. Furthermore, defective cilia formation in cultured POMC neurons limited axonal growth as well as dendrite formation and arborization, which would alter synaptic input organization onto POMC neurons and thus change the neuroelectrical properties as we observed in the electrophysiological study. Hence, disrupted ciliogenesis in the developing POMC neurons interferes with the proper organization of the neuronal circuits and exerts a lifelong adverse influence on the homeostatic regulation of food intake and energy expenditure.

Consistent with the findings in our cilia mutant models, a POMC-specific congenital deletion of retinitis pigmentosa GTPase regulator-interacting protein-1 like (Rpgrip1l), a ciliary protein that promotes ciliogenesis and mediates periciliary trafficking of leptin receptors, causes hyperphagic obesity^38^. In contrast, adult-onset Rpgrip1l depletion in POMC neurons had no effect on body weight^46^. Interestingly, POMC-specific Rpgrip1l mutants displayed enhanced axonal projections to PVH despite a reduction in POMC neuron numbers^46^. Taken together with our findings, developmental ciliary defects disrupt the normal maturation and/or wiring of POMC neurons and causes adulthood obesity although the changes in POMC neuronal circuits may be diverse depending on the cause of the ciliary defect.

Lysosomes are best known as the primary degradative compartment in eukaryotic cells^47^. In the lysosomal degradation process, macromolecules are transported to lysosomes via endocytic pathways or autophagy. In our study, POMC neurons with ciliary dysgenesis display the reduced lysosomal degradation of both autophagic substrates (LC3B and p62) and the endocytic substrate (DQ-BSA). In these cells, lysosomal mass and sizes are increased, which may be a compensatory change to the reduced lysosomal function. Conversely, the inhibition of lysosomal protein degradation by leupeptin administration significantly disrupts hypothalamic ciliogenesis. These data indicated a previously unexpected functional link between cilia and lysosomes.

Autophagy actively occurs in the hypothalamus in the early postnatal period^36^. In the previous studies, the inhibition of autophagy by depleting autophagy genes in POMC neurons inhibits POMC axonal innervation and increases fat mass and glucose intolerance in later life^36,48^. Indeed, our study show that defective ciliogenesis reduces autophagy flux without an impairment in autophagosome formation in POMC neurons. Thus primary cilia may modulate autophagic processes by controlling lysosomal degradation of autophagic vacuoles. During POMC circuit formation, many proteins would undergo lysosomal degradation to produce new proteins required for axon growth. Hypothalamic neurons undergoing axonogenesis may be thus in high demand for lysosomal proteolysis. In support of this possibility, inhibiting lysosomal protease activity with leupeptin from P8 to P13 significantly attenuated POMC axonal projections.

The postnatal leptin surge acts as an important developmental cue in the hypothalamic neurons by stimulating axonal projections and the circuit formation of ARH neurons^11,12^. These trophic actions of leptin depend on a functional leptin receptor LepRb^49^, but the details of the molecular mechanisms downstream of LepRb remain largely unknown. Our current study has shown that adequate ciliogenesis is critical for the tropic actions of leptin. Moreover, we show that postnatal leptin surge stimulates hypothalamic ciliogenesis, which may be a prerequisite for leptin-triggered circuit organization. As for the mechanism behind this phenomenon, our study shows that leptin lowers the lysosomal pH by increasing the proton-pump v-ATPase activity and promotes lysosomal degradation of autophagolysosome in a primary cilia-dependent manner. In line with our findings, leptin activates autophagy in non-neuronal cells and cancer cells^50,51^ and stimulates the autophagy/lysosome-mediated degradation of long-lived proteins in adipocytes^50^. Early postnatal leptin replacement completely reversed the short cilia phenotype, lysosomal dysfunction, and disruption of POMC neural circuits in the hypothalamus of ob/ob mice. In contrast, the same treatment in adult ob/ob mice rescued the reduction in hypothalamic ciliogenesis and lysosomal protein degradation but it failed to reverse disrupted POMC axonal projection. Hence, impaired ciliogenesis in developing POMC neurons may irreversibly disrupt the neural circuit organization.

Previous studies have demonstrated that maternal diets significantly influence the organization of hypothalamic feeding regulatory circuits via as yet unidentified mechanisms^44,45^. In our current study, maternal overnutrition and undernutrition during gestation and lactation profoundly inhibited early postnatal hypothalamic ciliogenesis in the offspring. Interestingly, abnormal maternal nutrition, which was restricted to the lactation period, caused comparable changes in hypothalamic cilia. Along with ciliary changes, pups raised by HFD- and LPD-fed dams exhibited lysosomal dysfunction and reduced axonal projection of hypothalamic POMC and non-POMC neurons. It is thus conceivable that a maternal HFD and LPD disrupt the hypothalamic neural circuit organization in the offspring through disrupted hypothalamic ciliogenesis and lysosomal dysfunction.

Notably, maternal diet-induced changes in hypothalamic cilia, lysosomes, and axonal growth phenocopy those caused by a leptin deficiency. As reported previously^44^, the plasma leptin levels were decreased by poor maternal nutrition during the early postnatal life. Moreover, daily leptin treatment during this period improved the maternal LPD-induced hypothalamic changes. These data suggest that a relative leptin deficiency may underlie maternal undernutrition-associated ciliary changes. In contrast, maternal HFD feeding exaggerates the postnatal leptin surge^45^, which may reflect leptin resistance. Supporting it, in our study, decreased hypothalamic ciliogenesis and POMC axonal growth in the offspring from HFD-fed mothers were not rescued by postnatal leptin replacement.

Collectively, our findings suggest that the primary cilia in hypothalamic neurons may serve as a critical node that bridges poor early-life nutritional conditions to adult-life obesity and metabolic disorders.

## Methods

### Cell culture

N1 hypothalamic neuronal cells were cultured in Dulbecco’s modified Eagle’s medium (DMEM) supplemented with 10% fetal bovine serum (FBS) and 1% penicillin/streptomycin.

### Mice

POMC-cre mice (Jackson Laboratory, #010714) were mated with either IFT88^f/f^ (Jackson Laboratory, #022409) or KIF3A^f/f^ mice (MGI, #2386464). POMC-cre/ERT2 mice (MGI, #5569339) were also mated with IFT88^f/f^ mice. Gene knockouts were induced by peritoneal injections of tamoxifen (75 mg/kg/day) into POMC-cre/ERT2;;IFT88^f/f^ mice in the either postnatal (P1–P14) or adult (P50–P55) life. IFT88^f/f^ controls received the same amount of tamoxifen. To generate mice with tdTomato-labeled POMC neurons, POMC-cre mice were mated with mice harboring a tdTomato reporter allele with an upstream *loxP*-flanked STOP cassette (Jackson Laboratory, #007909). C57BL/6J male mice at 7 weeks of age were purchased from Orient Bio (Seongnam, Korea). The ob/+ breeding pairs were obtained from the National Institute for Food and Drug Safety Evaluation (Choengju, Korea) to generate ob/ob mice. Mice were fed a standard CD (Samyang, Seoul, Korea) or HFD (58% fat; Research Diet, #D12331). C57 dams were fed a CD, HFD, or LPD (6% protein, Envigo, #TD90015) during the gestation and lactation periods or only for the lactation period. Animals were housed under a controlled temperature (22 ± 1 °C), humidity (55 ± 5%), and a 12-h light–dark cycle (lights on at 8 a.m.) with free access to food and water. All animal procedures were approved by the Institutional Animal Care and Use Committee of the Asan Institute for Life Science (Seoul, Korea).

### Metabolic phenotyping

Body weights, food intake levels, and body lengths (from the nostril to the tail) were monitored weekly between 9:00 a.m. and 10:00 a.m. with a digital weight scale at the indicated ages. Lean mass and fat mass were measured with dual X-ray absorptiometry (iNSiGHT VET DXA; OsteoSys, Seoul, Korea). Energy expenditure was measured with a comprehensive laboratory animal monitoring system (Columbia Instrument). For the glucose tolerance test, d-glucose (1 g/kg, Sigma) was administrated via an oral route under overnight fasting conditions. For the insulin tolerance test, insulin (Humulin-R^®^ 0.25 U/kg, Eli Lilly) was injected into the peritoneum in overnight fasted mice. Blood samples were obtained from the tail vein at the indicated time points after injections for glucose measurements using a glucometer (ACCU-CHEK^®^, Aviva Plus System).

### Antibodies

The primary antibodies used for immunostaining and immunoblotting were as follows: AC3 (Santa Cruz, #sc-588), ARL13B (Proteintech, #17711-1-AP), BrdU (Novus, #NB500-169), β-END (Phoenix Pharmaceuticals, #H-022-33 or Abcam, #Ab32893), IFT88 (Proteintech, #13967-1-AP), LepRb (R&D Systems, #AF497), LAMP1 (BD Pharmingen, #553792), LC3B (Abcam, #Ab51520), MAP2 (Abcam, #Ab5392), Neurofilament (Biolegend, #837904), NPY (Abcam, #Ab6173), p62 (Abcam, #Ab91526), and TFEB (Bethyl Laboratories, #A303-673A).

### Immunostaining

Cardiac perfusion was performed in mice with 50 ml saline followed by 50 ml 4% paraformaldehyde (PFA) under the anesthesia induced with 40 mg/kg Zoletil^®^ and 5 mg/kg Rompun^®^. Whole brains were collected, post-fixed with 4% PFA at 4 °C for 16 h, and dehydrated in 30% sucrose solution until the brain sank to the bottom of the container unless described otherwise. For the staining of NPY soma and LC3B, brains were fixed by cardiac perfusion with 1% PFA without post-fixation. For the staining of β-END axonal fibers, animals were perfused with 4% PFA dissolved in borate buffer (38 g/l, pH 9.5) and post-fixed in 20% sucrose–4% PFA–borate solution at 4 °C for 5 h. Coronal brain slices including the hypothalamus were sectioned at 20–30-μm thickness using a cryostat (Leica). One out of every five slices (in adults) and one out of every three slices (in embryos and neonates) were collected and stored at −70 °C. Hypothalamic slices were blocked with 3% donkey serum in phosphate-buffered saline (PBS) (NPY, LAMP1, p62, LC3B, LepRb), 3% goat serum in PBS (BrdU), and 3% BSA in 0.5% PBST (β-END, AC3, TFEB) for 1 h at room temperature (RT). For BrdU staining, brain slices were incubated with 2 N HCl for 30 min and permeabilized in 0.1% PBST for 5 min before blocking. For in vitro immunostaining, cells were fixed with 4% cold PFA for 15 min and then blocked with 1% BSA at RT for 1 h. Subsequently, slices or cells were incubated with primary antibodies at 4 °C for 16 h and then at RT for 1 h with the following dilutions: AC3 (1:1000), ARL13B (1:1000), BrdU (1:400), β-END (1:1500 for Abcam antibody, 1:1000 for Pheonix antibody), LAMP1 (1:500), LC3B (1:200), MAP2 (1:200), Neurofilament (1:1000), NPY (1:1,000), LepRb (1:100), p62 (1:200), and TFEB (1:500). After washing, the slides were incubated with the appropriate Alexa-Fluor 488-, 555-, 546-, 633-, or 647-conjugated secondary antibodies (1:1000) at RT for 1 h. For nuclear staining, slides were counterstained with 4,6-diamidino-2-phenylindole (DAPI; 1:10,000) for 10 min before mounting. Immunofluorescence was imaged using confocal microscopy (Carl Zeiss 780). Quantification of fluorescence intensity and cell counting were performed using Image J (version 1.52p, NIH), Photoshop CS6 (version 13.0x64, Adobe Systems), or Imaris (version 8.1.2, Build 36825 for x64, Oxford Instruments). The average fluorescent intensity of approximately 100 cells was analysed from 3 to 5 hypothalamic sections per mouse or 5 microscopic fields per well.

### Cilia analysis

For quantitation of the primary cilia lengths, cilia images were taken by *Z*-stacking using a confocal microscope (Carl Zeiss) and analysed with the ZEN microscope software (version 2.1 blue edition, Carl Zeiss). The average ciliary lengths of POMC, non-POMC, or ARH neurons (~100 each) were analysed. The ciliated cell percentage was determined by the number of cilia divided by the DAPI counts.

### POMC neuron numbers and axonal projection analysis

The numbers of β-END^+^ or tdTomato^+^ POMC neurons were counted in the 3–5 slices per mouse and then multiplied by the number of hypothalamic slices. Quantitative analysis of the POMC axonal projection intensity in the PVH, DMH, and LH areas was conducted by Image J. Each image plane was binarized, so as to isolate labeled fibers from the background. After adjustment of the brightness and contrast and elimination of background, we drew the region of interest (ROI) using the tool bar and measured the density of immunoreactivity in this ROI. The measurements of NPY neuron numbers and axonal projection were performed in the same way as mentioned above.

### Neurogenesis and neuronal cell death

To examine the embryonic neurogenesis of POMC neurons, BrdU (100 mg/kg, Sigma) was injected daily into the peritoneum of dams at E10.5, E11.5, and E12.5. Brains from the POMC-cre;;IFT88^f/f^ and IFT88^f/f^ offspring were collected at P21. We also accessed post-weaning POMC neurogenesis by daily injection of BrdU (100 mg/kg) for 3–6 weeks and by collecting brains at week 8. The β-END/BrdU dual staining was performed as described in the immunostaining section. BrdU-positive cell numbers in the ARH were counted in the brain sections (three slices per animal). To assess POMC neuron death, hypothalamic slices were subjected to β-END staining and then TUNEL staining using a commercial kit (Roche Applied Science).

### Axon and dendrite outgrowth

Primary neuron cultures were prepared from the hypothalamus of POMC-cre;;IFT88^f/f^;;tdTomato embryos and POMC-cre;;tdTomato embryos at E21. Tissues were chopped to small pieces in pre-warmed Hank’s Balanced Salt Solution media containing 0.002% DNase 1 (Sigma) and incubated in high-glucose DMEM (25 mM, Gibco) containing 0.01% trypsin for 15 min at 37 °C and centrifuged at 159 × *g* for 5 min at 4 °C. The supernatant was removed and 10% FBS-containing neurobasal (NB) media was then added to the pellet. The pellets were gently resuspended by pipetting and centrifuged at 159 × *g* for 5 min at 4 °C. To remove debris, washing steps were repeated three times. After washing, cells were plated in 6-well dishes and cultured in NB medium with B27 supplement (Thermo Fisher Scientific) for up to 21 days. Triple staining of tdTomato, MAP2, and neurofilament was conducted to access axonal and dendritic outgrowth of tdTomato-labeled POMC neurons on 7, 14, and 21 days in vitro as described in the “Immunostaining” section. Sholl analysis was used to examine dendrite arborization. We also evaluated the formation of the neuronal process in vivo by counting the tdTomato^+^ primary neuronal process of POMC neurons in 3-week-old mice.

### Electrophysiology

Whole-cell patch-clamp recordings from POMC neurons were performed in hypothalamic slice preparations. The brain slices including the ARH were obtained from POMC-cre;;tdTomato, POMC-cre;;KIF3A^f/f^;;tdTomato, and POMC-cre;;IFT88^f/f^;;tdTomato mice at 5–14 weeks. The pipette solution for whole-cell recording contained 120 mM Cs-gluconate, 10 mM CsCl, 10 mM HEPES, 5 mM EGTA, 1 mM CaCl_2_, 1 mM MgCl_2_, 2 mM MgATP, and 0.03 mM Alexa Fluor 488 hydrazide dye (pH 7.3). Electrophysiological signals were recorded using an Axopatch 700B amplifier (Molecular Devices) that was low-pass filtered at 1 kHz and analysed offline on a PC with pCLAMP programs (Molecular Devices). Recording electrodes had resistance of 2.5–5 MΩ when filled with the Cs-gluconate internal solutions. mEPSCs were recorded as inward currents at a holding potential of −60 mV, and mIPSCs were recorded as outward currents at a holding potential of −10 mV.

### Gene knockdown

For AAV-mediated knockdown of the IFT88 gene, AAV-DJ-EF1a-DIO-TATAlox-DSE-EYFP-shIFT88 (AAV-DIO-shIFT88-GFP) and AAV-Cre-GFP constructs were generated at the Virus Core of Korean Institute for Science and Technology. The sequence of mouse IFT88 shRNA was 5’-TTTGGAGCTTATTACATTGATATTCAAGAGATATCAATGTAATAAGCTCCAATTTTTTG-3’. A 1:1 mixture of AAV-DIO-shIFT88-GFP and AAV-Cre-GFP (1 × 10^12^ genome copies (GC)/ml each, 500 nl volume each side) was microinjected into the bilateral mediobasal hypothalamus of ob/ob neonates at P7 (stereotaxic coordinates: 0.7 mm caudal to the bregma, 0.1 mm to the sagittal sinus, and 4.4 mm ventral to the sagittal sinus). Mice showing an off-target viral injection were regarded as controls. N1 cells were cultured in 12-well plates and transfected with a 1:1 mixture of AAV-DIO-shIFT88-GFP and AAV-cre-GFP (1 × 10^9^ GC/ml each). Controls were transfected with AAV-GFP (2 × 10^9^ GC/ml). Successful AAV injections were confirmed by GFP expression and cilia staining.

### Immunoblotting

Mouse hypothalamic protein lysates (20 µg protein) were separated by 10% (w/v) sodium dodecyl sulfate-polyacrylamide gel electrophoresis and transferred to polyvinylidene difluoride membranes (Bio-Rad). Following blocking in 5% skim milk, the membranes were incubated overnight at 4 °C with primary antibodies against IFT88 (1:1000). Blots were developed using a horseradish peroxidase-linked secondary antibody (1:1000) and the chemiluminescent detection system (PerkinElmer). Bands were quantified with a densitometer (VersaDoc Multi Imaging Analyzer System; Bio-Rad) and normalized to the density of β-actin.

### Assessment of autophagy

To assess autophagy flux, either saline or leupeptin (40 mg/kg, Sigma) was injected in the peritoneum of 10-day-old mice at 6 and 24 h prior to sacrifice. Brains were collected following cardiac perfusion, sliced, and subjected to LC3B and β-END dual staining. The average LC3B puncta number in 100 POMC neurons per mouse was determined. And then the autophagy flux was calculated as follows: [The LC3B puncta number in leupeptin-injected mice] − [the average value of saline-injected mice with the same genotype]. We also evaluated the autophagy in cilia-defective POMC neurons by p62 immunostaining described in the “Immunostaining” section.

### Lysosomal mass and function

Lysosomal protein degradation activity was determined using DQ-red BSA (Thermo Fischer Scientific), which was administered at 2 μl into the third cerebroventricle of the animals at 24 h prior to sacrifice. Brains were collected after 4% PFA cardiac perfusion, post-fixed, and dehydrated in sucrose solution as described in the “Immunostaining” section. In vitro experiment, cells were treated with DQ-BSA (10 mg/ml) for 1 h before fixation. Red fluorescence was observed under confocal microscopy, and the DQ-BSA fluorescent intensity in each cell was measured. The average values of 100 cells per mouse or per well were presented. Lysosomal mass (total LAMP1 fluorescence intensity) and lysosomal size (the size of LAMP1^+^ granules) were analysed with LAMP1 staining. To monitor the hypothalamic lysosomal biogenesis, we assessed TFEB activation in the ARH cells by examining nuclear TFEB expression using the TFEB and DAPI double staining. In the vitro experiments, N1 cells were treated with LysoTracker or LysoSensor probes (Thermo Fischer Scientific, 1:1000) and Fl-albumin (Invitrogen, #A34787, 2 μM) for 30 min at 37 °C prior to fixation. The fluorescent intensity of LysoTracker or LysoSensor and Fl-albumin^+^ puncta numbers per cell were measured in 100 cells per well.

### V-ATPase activity

v-ATPase activity was measured using a commercial assay kit (Abcam, #Ab234055). Briefly, N1 cells were pretreated with bafilomycin (1 μM) for 1 h and then treated with leptin (100 nM) for 30 min prior to cell harvest. In the other set of experiments, cells were transfected with IFT88 small inhibitory RNA (siRNA) (Thermo Fisher, #MSS211712) and control siRNA (Dharmacon, #D-001910-10-05) 48 h prior to the leptin treatment.

### Maternal diet study

To study the effects of maternal diets on hypothalamic ciliogenesis in pups, dams were fed with a CD, HFD, or LPD from mating or from delivery until weaning. The litter size of each diet group was matched to exclude overfeeding or underfeeding (six per dam). Pups were sacrificed at P14 for the cilia analysis and DQ-BSA studies at P11. In separate analysis, pups were breastfed by dams receiving CD, HFD, or LPD feeding, injected with leptin from P4 to P13, and sacrificed at P14 for the hypothalamic cilia and lysosomal analysis. Plasma leptin concentrations in the offspring were measured at P10 by using leptin enzyme-linked immunosorbent assay (Invitrogen, #KMC2281).

### Leptin studies

To study the effects of leptin on hypothalamic ciliogenesis, lysosomal function, or neuronal circuit formation, ob/ob pups received intraperitoneal saline or leptin treatment (10 mg/kg/day, R&D Systems) at the indicated time period. Saline-injected ob/+ neonates were used as controls. Conversely, to inhibit postnatal leptin action, C57 neonates received intraperitoneal injections of the leptin antagonist SHLA (3 mg/kg/day, MyBioSource). For the shIFT88-AAV study, ob/ob pups were injected with AAV at P7 as described in the “Gene knockdown” section and then received leptin treatment from P10 to P14.

### Leptin receptor expression and periciliary trafficking

For the determination of hypothalamic leptin receptor expression in the offspring, the hypothalamic tissue blocks were collected at P14 and immediately frozen in liquid nitrogen. RNA was extracted and subjected to real-time PCR analysis for hypothalamic *Leprb* expression analysis using the primers (Supplementary Table 1). Expression of *Leprb* mRNA was normalized to that of glyceraldehyde-3-phosphate dehydrogenase (*Gapdh*). The comparative C_T_ method was used for the relative quantification of gene expression. To evaluate the periciliary trafficking of leptin receptors in hypothalamic neurons, the hypothalamic slices were collected at P15 from ob/ob pups that were received leptin alone or leptin + shIFT88-GFP-AAV injection and subjected to AC3/LepRb/GFP triple staining. The numbers of ARH cells with periciliary accumulation of leptin receptors were counted in three hypothalamic slices per mouse.

### Statistics

All data values are presented as mean ± standard error of the mean (SEM). Statistical analyses were performed using SPSS version 24 (IBM Analytics) or Prism version 7.0 (GraphPad). The sample sizes were selected based on previous studies that used similar methodologies. Statistical significance among the groups was tested using one-way, two-way, or repeated measures analysis of variance followed by post hoc least significant difference test or a two-sided Student’s *t* test if appropriate. Statistical significance was defined by a *p* < 0.05.

### Reporting summary

Further information on research design is available in the Nature Research Reporting Summary linked to this article.

## Supplementary information

Supplementary Information

Reporting Summary

## Data Availability

All data supporting the findings are presented within this paper and its supplementary information. Data originated in this study have also been deposited here: 10.6084/m9.figshare.13077764.v1. Additional information is available upon reasonable request to the corresponding author. Source data are provided with this paper.

## Source data

Source data
