# Supplementary material for: Primary cilia mediate early life programming of adiposity through lysosomal regulation in the developing mouse hypothalamus
Source: Nat Commun. 2020 Nov 13;11:5772. doi: 10.1038/s41467-020-19638-4 (PMC7666216; doi:10.1038/s41467-020-19638-4)
Supplement: Supplementary file 1 — Supplementary Information [file 41467_2020_19638_MOESM1_ESM.pdf]

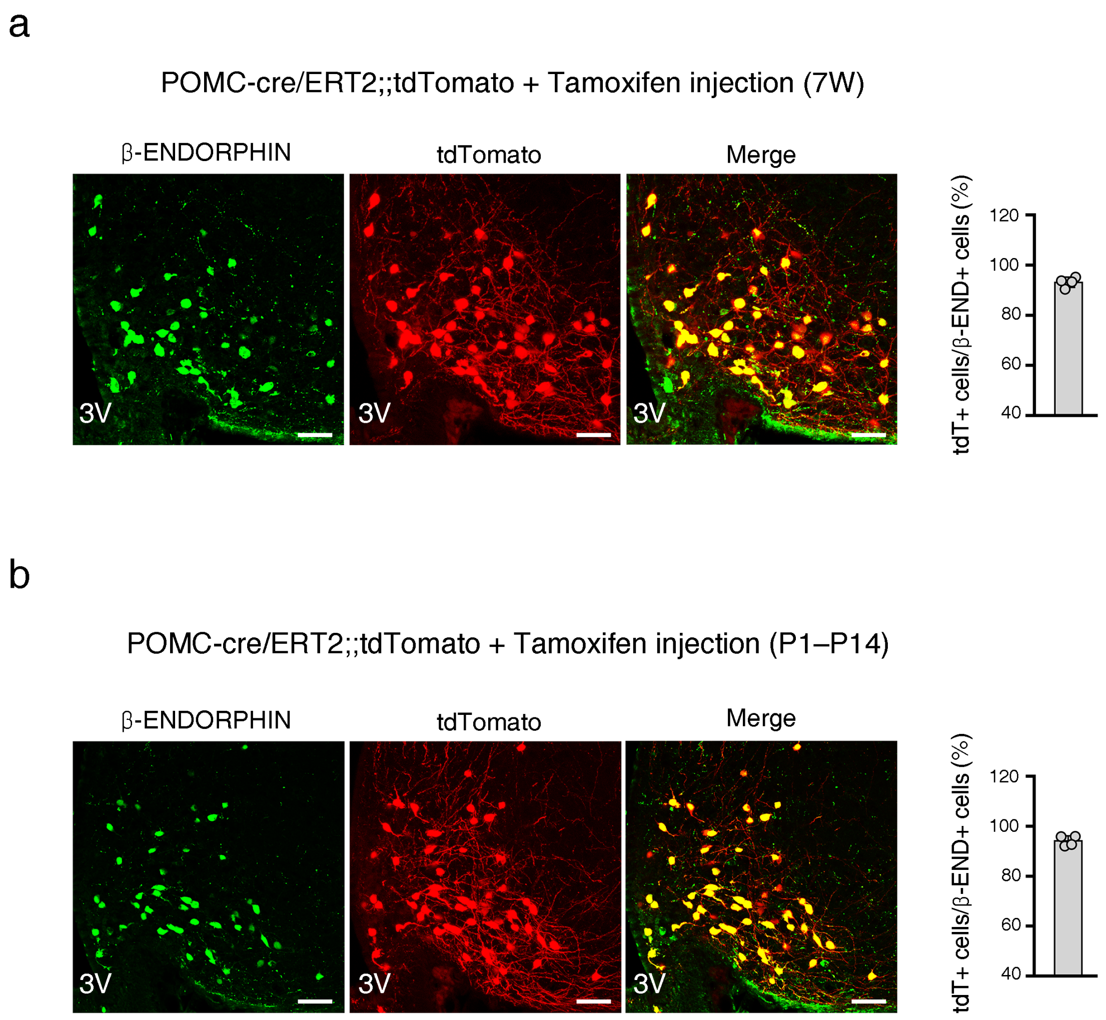

Supplementary Fig. 1 Confirmation of cre-lox recombination

Immunostaining of β-ENDORPHIN (β-END) and tdTomato (tdT) double immunostaining in the ARH of POMC-cre/ERT2;;tdTomato mice to confirm the successful cre-lox recombination induced by tamoxifen injections (n = 4). Tamoxifen was injected at 7 weeks (a) or during P1-P14 (b).
The graphs depict the percentages of tdTomato-expressing cells among β-END^{+} POMC neurons.
Data are presented as a means ± SEM. Scale bars: 50 µm

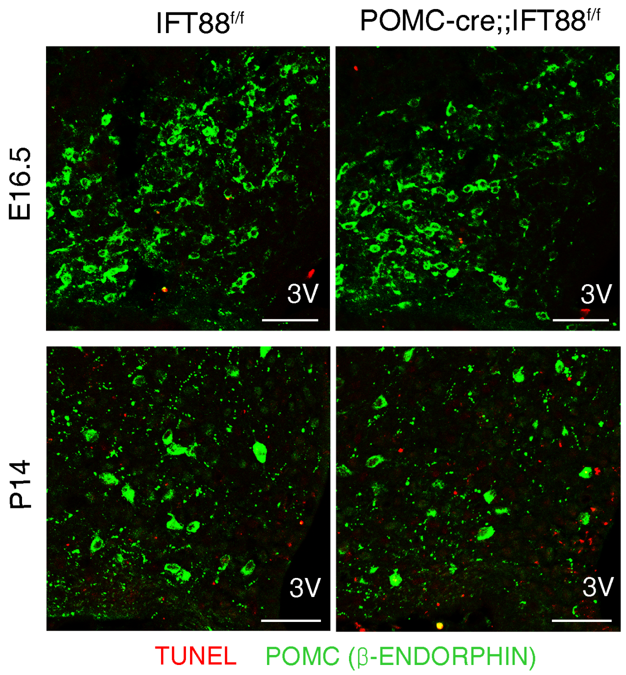

Supplementary Fig. 2 Assessment of cell death in POMC neurons with embryonic ciliary dysgenesis

Representative images of TUNEL and POMC (β-ENDORPHIN) double staining in the ARH of POMC-cre;;IFT88$^{f/f}$ mice and IFT88$^{f/f}$ mice at E16.5 and P14 (n = 3).

Scale bars: 50 μm. 3V: 3rd ventricle.

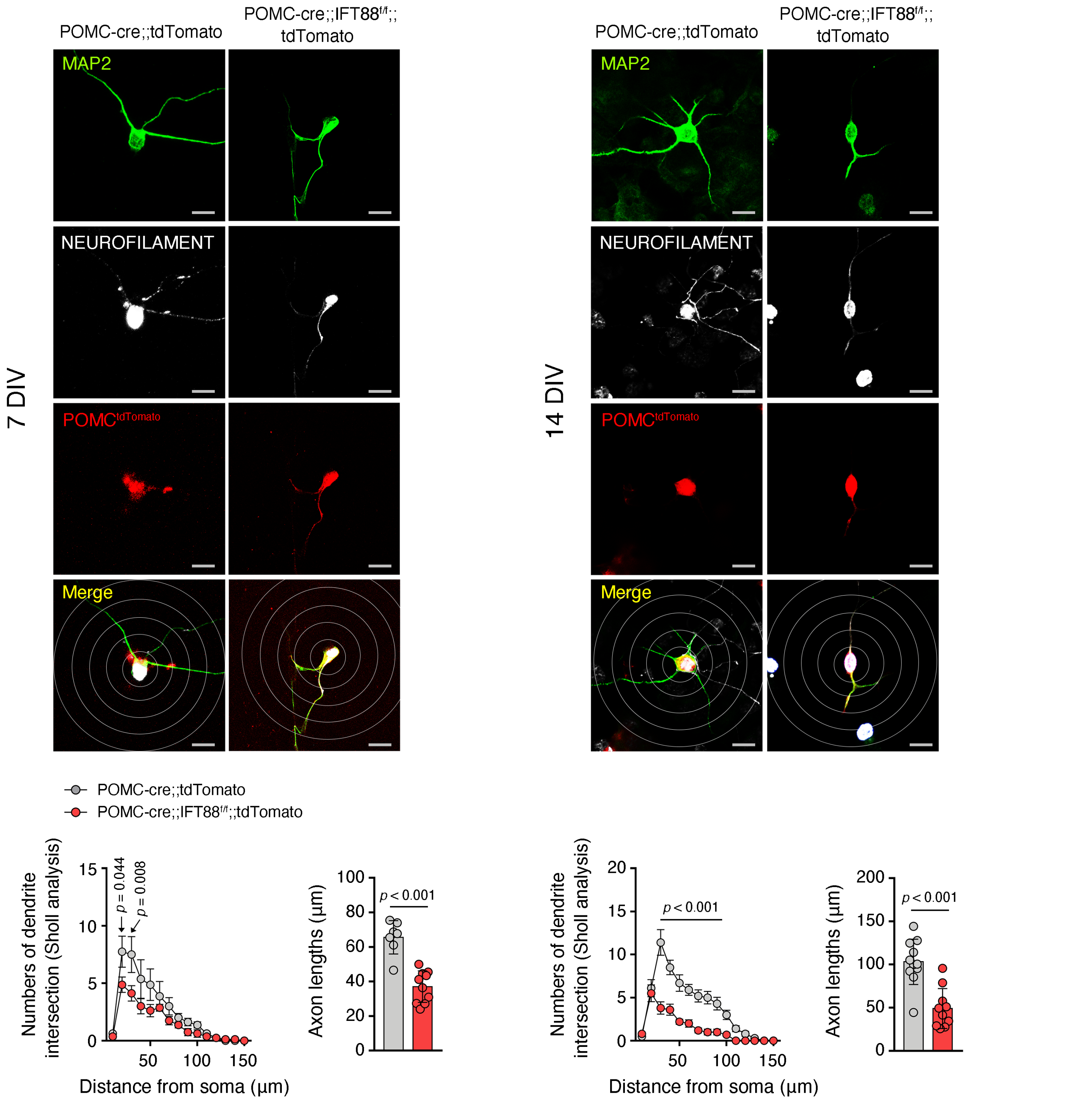

Supplementary Fig. 3 Axon and dendrite outgrow in the cultured POMC neurons with defective ciliogenesis

Representative images of MAP2 (dendrite marker), NEUROFILAMENT (axon marker) and tdTomato (POMC) triple staining in primary cultured neurons obtained from POMC-cre;;IFT88$^{f/f}$;;tdTomato embryos and POMC-cre;;tdTomato embryos. Cells are culured for 7 and 14 days in vitro (DIV) before analysis.

Sholl analysis was conducted for evaluation of dendritic branching in 8-10 POMC neurons.

The full lengths of axons were measured for 7-11 POMC neurons.

Data are presented as a means ± SEM. Statistics was performed using one-sided two-way ANOVA followed by post hoc LSD test (for dendritic branching) and two-sided Student's *t* test (for axon growth).

Scale bars: 20 μm.

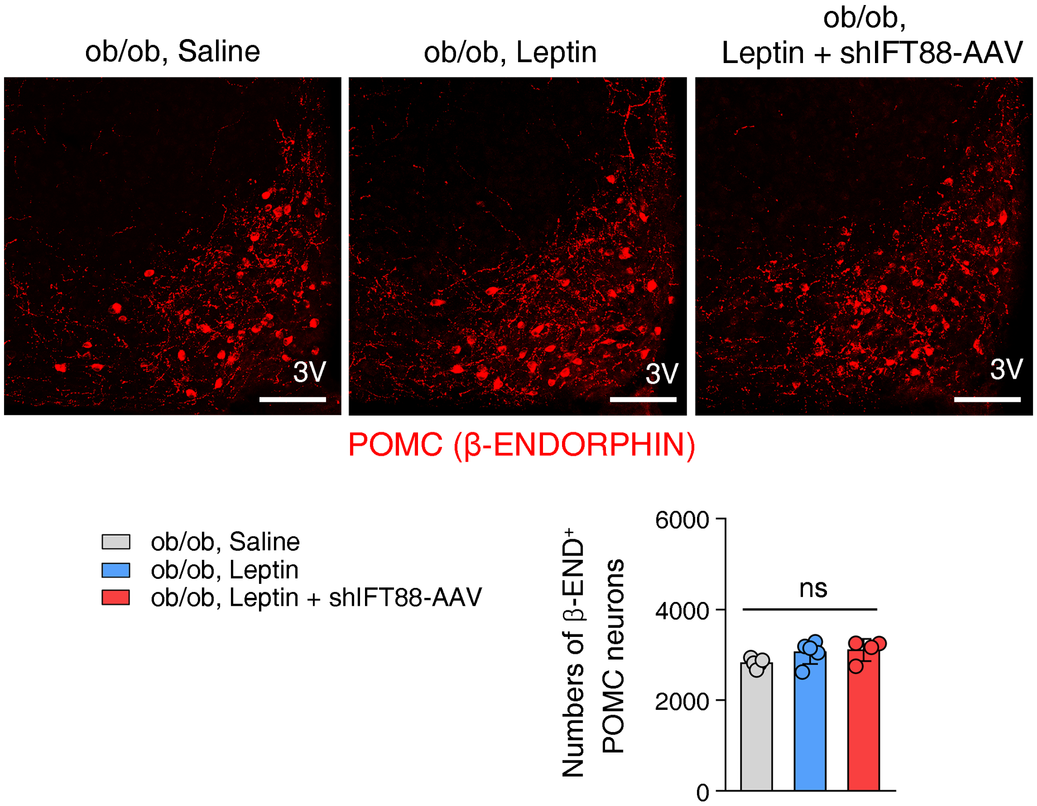

Supplementary Fig. 4 POMC neuron numbers in ob/ob neonates

β-ENDORPHIN immunohistochemistry in the ARH of ob/ob mice that received saline, leptin, or leptin + shIFT88-AAV (n = 5 for the ob/ob saline group and the ob/ob leptin group, n = 4 for the ob/ob leptin + shIFT88-AAV group). Data are presented as a means ± SEM.

Statistics performed using one-sided one-way ANOVA followed by post hoc LSD test.

ns: not significant. Scale bars: 50 µm.

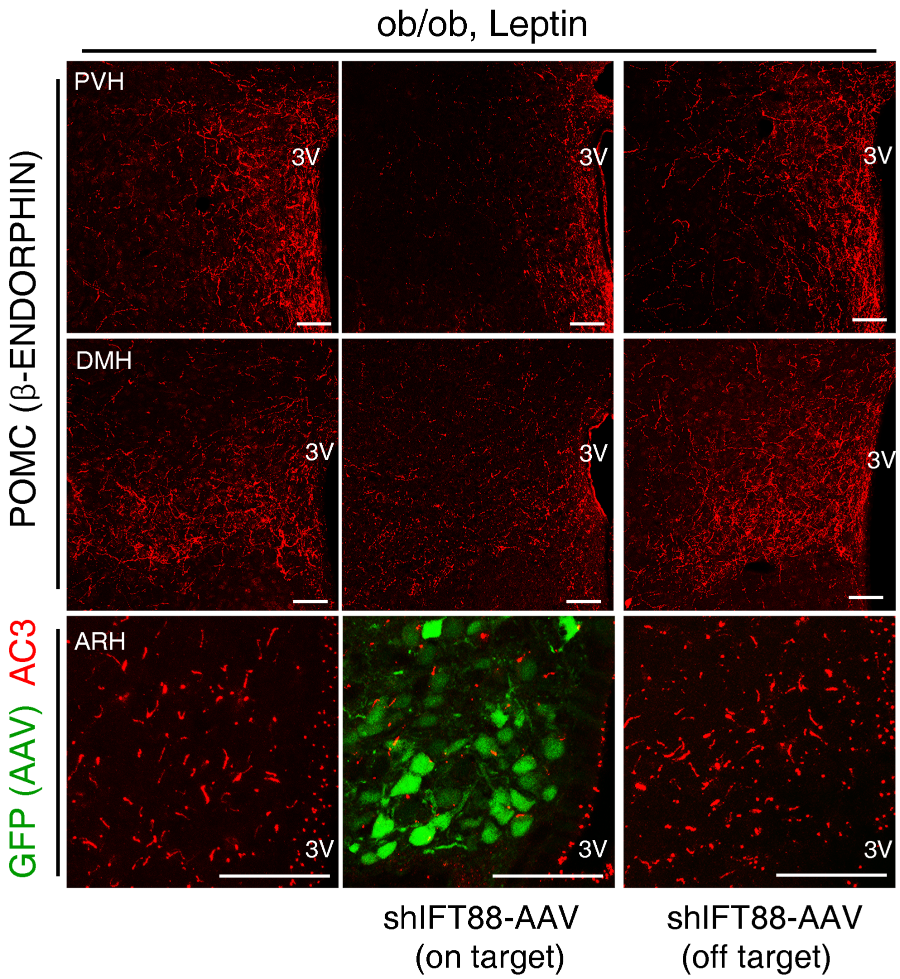

Supplementary Fig. 5 Comparison of POMC axonal projection and ARH ciliogenesis between shIFT88-GFP-AAV on target and off target groups

Representative images of β-ENDORPHIN immunohistochemistry and AC3 (cilia)/GFP double staining in 2-week-old ob/ob mice that received leptin alone or leptin + shIFT88-GFP-AAV.

Off target group was used as a control for shIFT88-GFP-AAV on target group ($n = 5$).

Scale bars: 50 μm. 3V: 3rd ventricle.

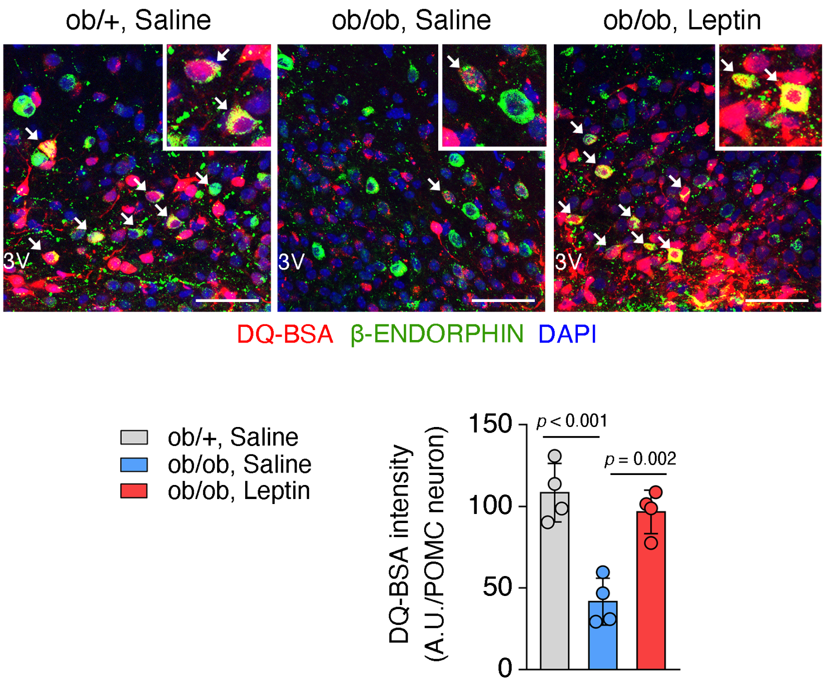

Supplementary Fig. 6 Lysosomal protein degradation in hypothalamic neurons of ob/ob mice with adulthood leptin treatment

DQ-BSA and β-ENDORPHIN double staining in the hypothalamic ARH in 7 week-old ob/+ mice, ob/ob mice, and ob/ob mice with adulthood leptin replacement (10 mg/kg/day for 7 days before sacrifice) (n = 4). The average values of DQ-BSA intensity in 100 β-END^{+} POMC neurons per mouse are presented. Arrows indicate DQ-BSA^{+} POMC neurons.

Data are shown as a means ± SEM. Statistics performed using one-sided one-way ANOVA followed by post hoc LSD test. Scale bars: 50 µm. 3V: 3rd ventricle.

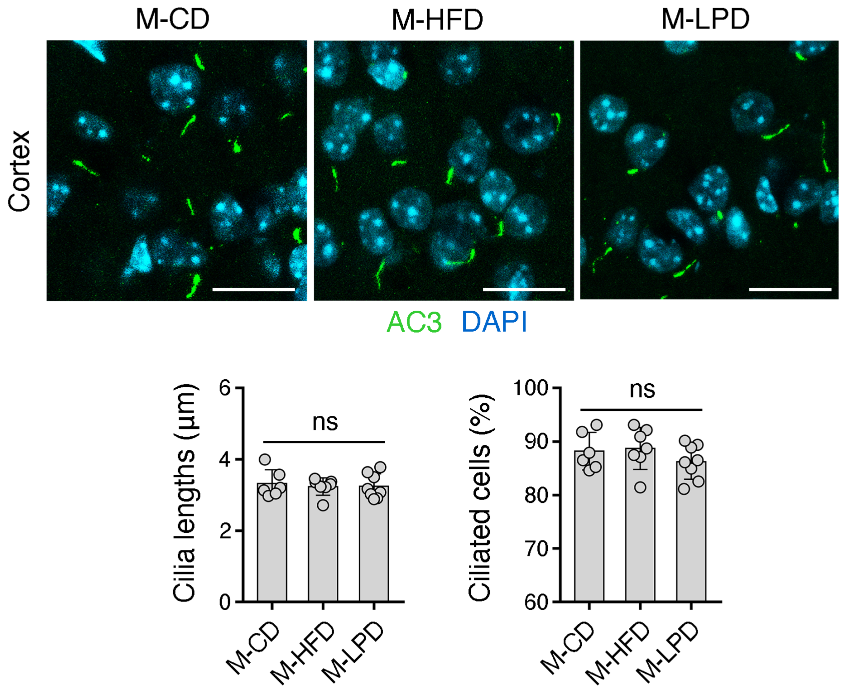

Supplementary Fig. 7 Ciliogenesis in the cortex of neonates during maternal diet manipulation
Cilia (AC3) staining in the cortex (parietal lobe) of offspring at P14 that were nourished by dams on a normal chow diet (M-CD), 60% high fat diet (M-HFD) or 6% low protein diet (M-LPD) during gestation and lactation ($n = 6$ for M-CD, $n = 7$ for M-HFD, and $n = 8$ for M-LPD). The average lengths of 100 cilia and the ciliated cell percentage per mouse are presented.
Data are presented as a means ± SEM. Statistics was analysed using one-sided one-way ANOVA followed by post hoc LSD test. ns: not significant. Scale bars: 20 µm.

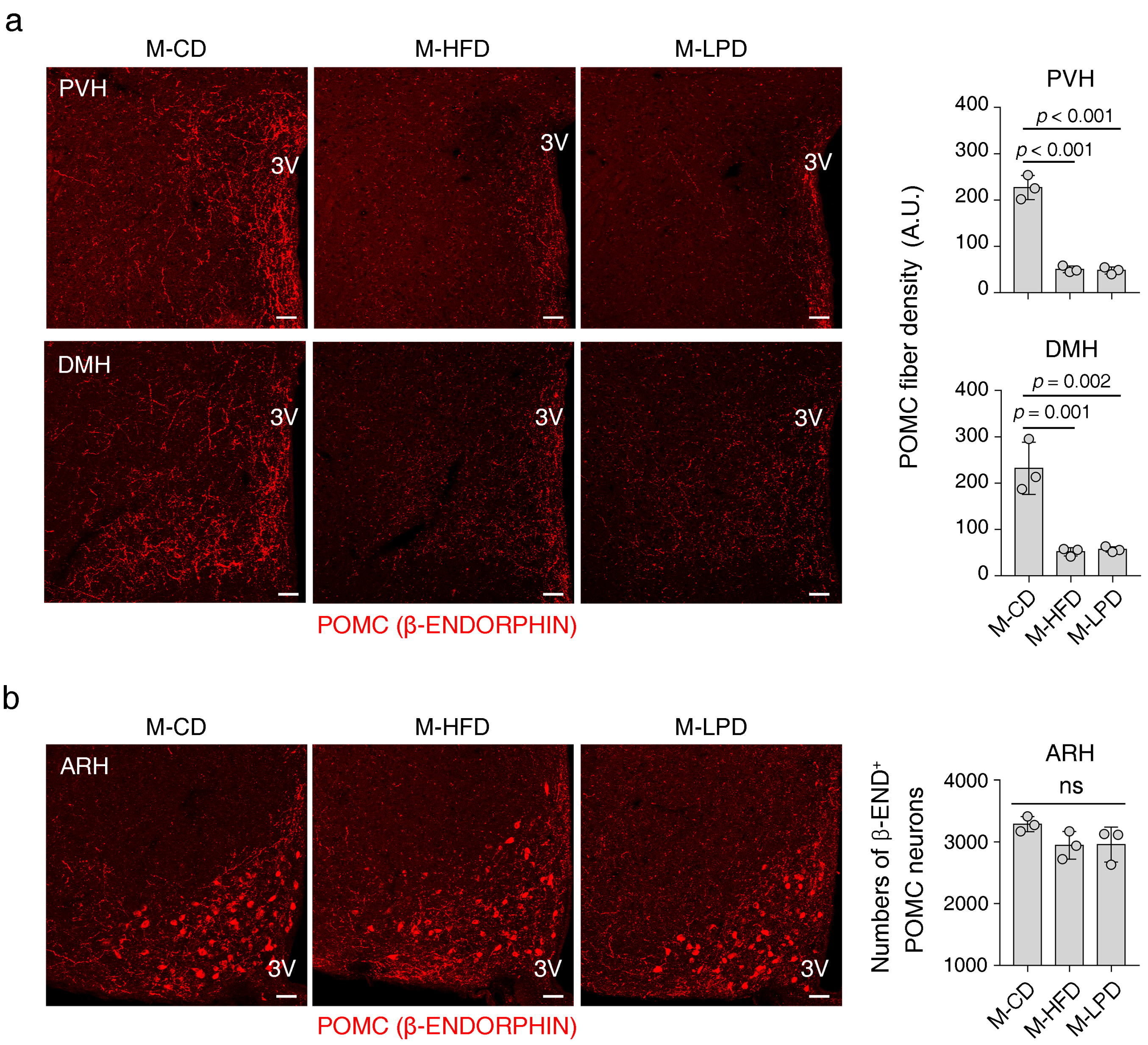

Supplementary Fig. 8 POMC fiber density and neuron numbers in the pups nourished by LPD- or HFD-fed dams

β-ENDORPHIN immunohistochemistry in the PVH, DMH (a), and ARH (b) of offspring at P14 that were nourished by dams on a normal chow diet (M-CD), high fat diet (M-HFD), or low protein diet (M-LPD) during gestation and lactation ($n = 3$).

Data are presented as a means ± SEM. Statistics was analysed using one-sided one-way ANOVA followed by post hoc LSD test. ns: not significant. Scale bars: 50 µm.

Supplementary Table 1 Primer sequences used for real time PCR analysis

Gene	Forward	Reverse
Leprb	5’-CCAAACAATGCCTCGGCTTT-3’	5’-CCTTGCTCATTCCCAAAGCA-3’
Gapdh	5’-CCTGTTGCTGTAGCCGTAT-3’	5’-ACTCTTCCACCTTCGATGC-3’
