# Supplementary material for: Primary cilia mediate early life programming of adiposity through lysosomal regulation in the developing mouse hypothalamus
Source: Nat Commun. 2020 Nov 13;11:5772. doi: 10.1038/s41467-020-19638-4 (PMC7666216; doi:10.1038/s41467-020-19638-4)
Supplement: Supplementary file 2 — Reporting Summary [file 41467_2020_19638_MOESM2_ESM.pdf]

nature research

Corresponding author(s): Min-Seon Kim

Last updated by author(s): Oct 12, 2020

Reporting Summary

Nature Research wishes to improve the reproducibility of the work that we publish. This form provides structure for consistency and transparency in reporting. For further information on Nature Research policies, see our Editorial Policies and the Editorial Policy Checklist.

Statistics

For all statistical analyses, confirm that the following items are present in the figure legend, table legend, main text, or Methods section.

n/a	Confirmed	
☐	☒	The exact sample size (*n*) for each experimental group/condition, given as a discrete number and unit of measurement
☐	☒	A statement on whether measurements were taken from distinct samples or whether the same sample was measured repeatedly
☐	☒	The statistical test(s) used AND whether they are one- or two-sided<br>*Only common tests should be described solely by name; describe more complex techniques in the Methods section.*
☒	☐	A description of all covariates tested
☒	☐	A description of any assumptions or corrections, such as tests of normality and adjustment for multiple comparisons
☐	☒	A full description of the statistical parameters including central tendency (e.g. means) or other basic estimates (e.g. regression coefficient) AND variation (e.g. standard deviation) or associated estimates of uncertainty (e.g. confidence intervals)
☐	☒	For null hypothesis testing, the test statistic (e.g. F, t, r) with confidence intervals, effect sizes, degrees of freedom and P value noted<br>*Give P values as exact values whenever suitable.*
☒	☐	For Bayesian analysis, information on the choice of priors and Markov chain Monte Carlo settings
☒	☐	For hierarchical and complex designs, identification of the appropriate level for tests and full reporting of outcomes
☐	☒	Estimates of effect sizes (e.g. Cohen's d, Pearson's r), indicating how they were calculated

Our web collection on statistics for biologists contains articles on many of the points above.

Software and code

Policy information about availability of computer code

Data collection	ZEN microscope software (version 2.1 blue edition, Carl Zeiss)
Data analysis	Image analysis:<br>ZEN microscope software (version 2.1 blue edition, Carl Zeiss),<br>Photoshop (CS6, Ver 13.0 x64),<br>Image J (Version 1.52p),<br>Imaris (Ver 8.1.2, Build 36825 for x64)<br><br>Data analysis:<br>Prism 7.0 (GraphPad)<br>SPSS version 24 (IBM Analytics)

For manuscripts utilizing custom algorithms or software that are central to the research but not yet described in published literature, software must be made available to editors and reviewers. We strongly encourage code deposition in a community repository (e.g. GitHub). See the Nature Research guidelines for submitting code & software for further information.

Data

Policy information about availability of data

All manuscripts must include a data availability statement. This statement should provide the following information, where applicable:

- Accession codes, unique identifiers, or web links for publicly available datasets
- A list of figures that have associated raw data
- A description of any restrictions on data availability

All source data are available at https://doi.org/10.6084/m9.figshare.13077764.v1.

Field-specific reporting

Please select the one below that is the best fit for your research. If you are not sure, read the appropriate sections before making your selection.

- [x] Life sciences
- [ ] Behavioural & social sciences
- [ ] Ecological, evolutionary & environmental sciences

For a reference copy of the document with all sections, see nature.com/documents/nr-reporting-summary-flat.pdf

Life sciences study design

All studies must disclose on these points even when the disclosure is negative.

Sample size	No statistical method was used to predetermine sample size in animal experiments. Cell numbers of relative experiments in this study were determined based on our experience and previous studies in this field.
Data exclusions	No data were excluded in this study.
Replication	All experiments were repeated at least three times unless otherwise indicated, and all attempts at replication were successful.
Randomization	For leptin or leupeptin injection experiments, mice were selected according to their body weight to match the average body weights between groups. Randomization was not relevant to other experiments, but control and experimental samples were always processed in parallel to control covariates.
Blinding	Investigators were not blinded in conducting animal experiments or collecting samples for analysis. Where possible, researchers were blinded during data analysis.

Reporting for specific materials, systems and methods

We require information from authors about some types of materials, experimental systems and methods used in many studies. Here, indicate whether each material, system or method listed is relevant to your study. If you are not sure if a list item applies to your research, read the appropriate section before selecting a response.

Materials & experimental systems

n/a	Involved in the study	
[ ]	[x]	Antibodies
[ ]	[x]	Eukaryotic cell lines
[x]	[ ]	Palaeontology and archaeology
[ ]	[x]	Animals and other organisms
[x]	[ ]	Human research participants
[x]	[ ]	Clinical data
[x]	[ ]	Dual use research of concern

Methods

n/a	Involved in the study	
[x]	[ ]	ChIP-seq
[x]	[ ]	Flow cytometry
[x]	[ ]	MRI-based neuroimaging

Antibodies

Antibodies used

Anti-AC3, rabbit polyclonal (Santacruz, Cat#: sc-588;RRID: AB_630839)
Anti-IFT88, rabbit polyclonal (Proteintech, Cat#: 13967-1-AP;RRID: AB_2121979)
Anti-beta-ENDORPHIN, rabbit polyclonal (Phoenix pharmaceuticals, Cat#: H-022-33;RRID: AB_2314007)
Anti-beta-ENDORPHIN, goat polyclonal (Abcam, Cat#: Ab32893;RRID: AB_777375)
Anti-BrdU, rat monoclonal (Novus, Cat#: NB500-169;RRID: AB_10002608)
Anti-NPY, sheep polyclonal (Abcam, Cat#: Ab6173;RRID: AB_305341)
Anti-MAP2, chick polyclonal (Abcam, Cat#: Ab5392;RRID: AB_2138153)
Anti-NEUROFILAMENT, mouse monoclonal (BioLegend, Cat#: 837904;RRID: AB_2566782)
Anti-LC3B, rabbit polyclonal (Abcam, Cat#: Ab51520;RRID: AB_881429)

Anti-P62, rabbit polyclonal (Abcam, Cat#: 91526;RRID: AB_2050336)
Anti-LAMP1, rat monoclonal (BD Biosciences, Cat#: 553792;RRID: AB_2134499)
Anti-ARL13B, rabbit polyclonal (Proteintech, Cat#: 17711-1-AP;RRID: AB_2060867)
Anti-Leptin R, goat polyclonal (R&D, Cat#: AF497;RRID: AB_2281270)
Anti-TFEB, rabbit polyclonal (Bethyl, Cat#: A303-673A;RRID: AB_11204751)
Anti-rabbit secondary antibody, donkey polyclonal, Alexa-Flour 488-conjugated (Invitrogen, Cat#: A21206; RRID: AB_141708)
Anti-chicken secondary antibody, goat polyclonal, Alexa-Flour 488-conjugated (Invitrogen, Cat#: A11039; RRID: AB_2534096)
Anti-mouse secondary antibody, goat polyclonal, Alexa-Flour 647-conjugated (Invitrogen, Cat#: A21236; RRID: AB_2535805)
Anti-goat secondary antibody, donkey polyclonal, Alexa-Flour 633-conjugated (Invitrogen, Cat#: A21082; RRID: AB_2535739)
Anti-goat secondary antibody, donkey polyclonal, Alexa-Flour 488-conjugated (Invitrogen, Cat#: A11055; RRID: AB_142672)
Anti-rat secondary antibody, goat polyclonal, Alexa-Flour 488-conjugated (Invitrogen, Cat#: A11006; RRID: AB_141373)
Anti-goat secondary antibody, donkey polyclonal, Alexa-Flour 555-conjugated (Invitrogen, Cat#: A21432; RRID: AB_2535853)
Anti-sheep secondary antibody, donkey polyclonal, Alexa-Flour 488-conjugated (Invitrogen, Cat#: A11015; RRID: AB_141362)
Anti-rabbit secondary antibody, donkey polyclonal, Alexa-Flour 555-conjugated (Invitrogen, Cat#: A31572; RRID: AB_162543)
Anti-rat secondary antibody, goat polyclonal, Alexa-Flour 546-conjugated (Invitrogen, Cat#: A11081; RRID: AB_141738)

Validation

Antibody validation information can be found on manufacturers' website.
Anti-AC3, https://datasheets.scbt.com/sc-588.pdf
Anti-IFT88, https://www.ptglab.com/products/IFT88-Antibody-13967-1-AP.htm
Anti-beta-ENDORPHIN (rabbit), https://www.phoenixpeptide.com/products/view/Antibodies/H-022-33
Anti-beta-ENDORPHIN (goat), https://www.abcam.com/pomc-antibody-ab32893.html
Anti-BrdU, https://www.novusbio.com/products/bromodeoxyuridine-brdu-antibody-bu1-75-icr1-_nb500-169
Anti-NPY, https://www.abcam.com/neuropeptide-y-antibody-ab6173.html
Anti-MAP2, https://www.abcam.com/map2-antibody-ab5392.html
Anti-NEUROFILAMENT, https://www.biolegend.com/en-us/products/purified-anti-neurofilament-marker-pan-axonal-cocktail-12811
Anti-LC3B, https://www.abcam.com/lc3b-antibody-ab51520.html
Anti-P62, https://www.abcam.com/sqstm1--p62-antibody-ab91526.html
Anti-LAMP1, https://www.bdbiosciences.com/us/applications/research/intracellular-flow/intracellular-antibodies-and-isotype-controls/anti-mouse-antibodies/purified-rat-anti-mouse-cd107a-1d4b/p/553792
Anti-ARL13B, https://www.ptglab.com/products/ARL13B-Antibody-17711-1-AP.htm
Anti-Leptin R, https://www.rndsystems.com/products/mouse-leptin-r-antibody_af497
Anti-TFEB, https://www.bethyl.com/product/A303-673A?referrer=search

Eukaryotic cell lines

Policy information about cell lines

Cell line source(s)	Mouse: N1 hypothalamic cell-line (CEDARLANE) Cat#: CLU101
Authentication	N1 cell lines were authenticated by CEDARLANE. No additional authentication was performed.
Mycoplasma contamination	All cell lines tested negative for mycoplasma contamination.
Commonly misidentified lines (See ICLAC register)	No commonly misidentified cell lines were used in the study.

Animals and other organisms

Policy information about studies involving animals; ARRIVE guidelines recommended for reporting animal research

Laboratory animals

Mouse: C57BL/6J (Orient bio)
Mouse: POMC-cre (Jackson Laboratory, #010714)
Mouse: POMC-cre/ERT2 (MGI, #5569339)
Mouse: B6.129P2-IFT88tm1Bky/J (Jackson Laboratory, #022409)
Mouse: KIF3Af/f (Mouse Genome Informatics, #2386464)
Mouse: tdTomato-loxP:B6.Cg-Gt(ROSA)26Sortm9(CAG-tdTomato)Hze/J (Jackson Laboratory, #007909)
Mouse: C57BL/6-Lepem1hwl/Korl National Institute of Food and Drug safety Evaluation (NIFDS)

Detailed gender and age information of mice were specified for each experiment in the manuscript.

All animals were housed under a controlled temperature (22 ±1خ), humidity (55 ± 5%) and a 12 light-dark cycle (lights on at 8 AM) with free access to food and water.

Wild animals	This study did not involve wild animals
Field-collected samples	This study did not involve field-collected samples.
Ethics oversight	Animal experiments in this paper were compliant with all relevant ethical regulations regarding animal research, and were conducted under the approval of he Institution Animal Care and Use Committee of the Asan Institute for Life Sciences (Seoul, Korea)

under licence number 2016-11-132.

Note that full information on the approval of the study protocol must also be provided in the manuscript.
